# Health inequalities and trends in heart failure diagnosis in primary care in England, 2000–21: a national retrospective cohort data-linkage study

**DOI:** 10.1016/j.lanprc.2025.100060

**Published:** 2025-12

**Authors:** Claire A Lawson, Mohammad R Ali, Gerry P McCann, Iain Squire, Francesco Zaccardi, Muhammad Rashid, Kelly Barber, Riaz Alaei Kalajahi, Christopher A Miller, Rhys Williams, Andrew L Clark, Mark C Petrie, Clare J Taylor, Jocelyn M Friday, Nathalie Conrad, John G F Cleland, Kamlesh Khunti

**Affiliations:** a Division of Cardiovascular Sciences, School of Medical Sciences, University of Leicester and NIHR Leicester Biomedical Research Centre, Leicester, UK; b Leicester Real World Evidence Unit, University of Leicester, Leicester, UK; c Leicester Partnership Trust, Leicester, UK; d Division of Cardiovascular Sciences, School of Medical Sciences, Faculty of Biology, Medicine and Health, Manchester Academic Health Science Centre, University of Manchester, Manchester, UK; e Manchester University NHS Foundation Trust, Manchester, UK; f Wellcome Centre for Cell-Matrix Research, Division of Cell-Matrix Biology & Regenerative Medicine, School of Biology, Faculty of Biology, Medicine & Health, Manchester Academic Health Science Centre, University of Manchester, Manchester, UK; g Princess of Wales Hospital, Bridgend, UK; h Hull University Teaching Hospitals NHS Trust, Castle Hill Hospital, Hull, UK; i British Heart Foundation Cardiovascular Research Centre, School of Cardiovascular and Metabolic Health, British Heart Foundation Glasgow, Glasgow, UK; j Department of Applied Health Sciences, University of Birmingham, Birmingham, UK; k School of Health & Wellbeing, University of Glasgow, Glasgow, UK; l Department of Cardiovascular Sciences, KU Leuven, Leuven, Belgium; m Nuffield Department of Women’s and Reproductive Health, University of Oxford, Oxford, UK

## Abstract

**Background:**

Heart failure is often diagnosed during unplanned admission to hospital, which is associated with poor outcomes. The aim of this study was to examine the potential for early heart failure identification in primary care, trends in diagnostic practices, sociodemographic inequalities, and the association between diagnostic pathways and outcomes.

**Methods:**

We conducted a retrospective cohort study using the Clinical Practice Research Datalink. Adults aged 18 years or older with newly diagnosed heart failure were identified from linked primary care and hospital records in England between Jan 1, 2000, and March 31, 2021. From primary care records, we analysed heart failure indicators (ie, breathlessness, ankle swelling, and loop diuretic use) recorded up to 5 years before diagnosis and diagnostic investigations (ie, natriuretic peptide tests, echocardiography, and specialist review) recorded within the previous 6 months. Trends over time and differences among sociodemographic groups are reported. Associations between diagnosis timing, investigation use, location (inpatient *vs* outpatient) and the primary outcome of 1-year survival were assessed, with analyses restricted to Jan 1, 2015, to Dec 31, 2019. Associations were adjusted for age, sex, ethnicity, socioeconomic status, year of diagnosis, systolic blood pressure, BMI, cholesterol, smoking, comorbidities, and prescribed drugs at diagnosis.

**Findings:**

Among 412 173 new heart failure diagnoses (median age 78·0 years [IQR 69·0–85·0]), 194 175 (47·1%) were women and 217 998 (52·9%) were men. In 407 622 participants with ethnicity data, ethnicity was recorded as White for 375 808 (92·2%), south Asian for 11 644 (2·9%), Black for 6994 (1·7%), other or mixed for 3622 (0·9%), and unknown for 9554 (2·3%). Although 274 228 (66·5%) of 412 173 patients had previous heart failure indicators, diagnostic timing worsened over the study period (2000–04 to 2015–19), with a median lag time increasing from 16·4 months (IQR 1·6–45·7) to 35·4 months (6·4–54·7) and the proportion of inpatient diagnoses rising from 30 560 (33·9%) of 90 136 patients to 55 905 (46·8%) of 119 355 patients. Among 80 824 individuals with previous indicators suggestive of heart failure diagnosed between Jan 1, 2015, and Dec 31, 2019, only 10 079 (12·5%) patients underwent natriuretic peptide testing, 15 986 (19·8%) had echocardiography, 27 804 (34·4%) were referred to specialists, and 42 302 (52·3%) had no diagnostic investigations recorded in primary care. Women, individuals living in deprived quintiles, and those with multiple long-term conditions had up to five times longer delays, lower investigation rates, and a higher likelihood of hospital diagnosis. Delays (adjusted hazard ratio [HR] 1·15 [95% CI 1·10–1·20]), absence of investigations (1·89 [1·83–1·95]), and inpatient diagnosis (2·58 [2·50–2·66]) were all associated with higher mortality. Mortality was lowest among outpatients who underwent primary care investigations (571 [5·5%] of 10 469 patients) and highest among inpatients with long-term loop diuretic use but no previous investigation (5429 [33·0%] of 16 458 patients; adjusted HR 5·29 [95% CI 4·83–5·79]).

**Interpretation:**

Most patients with heart failure show early signs in primary care, yet few receive timely diagnostic evaluation. Delays, missed investigations, and inpatient diagnoses are associated with poor outcomes and care inequities.

**Funding:**

British Heart Foundation and National Institute for Health Research.

## Introduction

Heart failure is frequently diagnosed late, often only after patients are admitted to hospital with severe symptoms or complications.^1^^,^^2^ Many patients with heart failure die before the problem is diagnosed.^3^^,^^4^ The universal definition of heart failure is based on symptoms and signs, supported by objective evidence of cardiac dysfunction.^5^ Accordingly, the diagnosis of heart failure relies on patients reporting symptoms and clinicians observing signs, understanding their significance, and initiating appropriate investigations. Breakdowns in the diagnostic process can occur at any of these stages.

**Research in context**

**Evidence before this study**

Heart failure is frequently diagnosed during unplanned admission to hospital, often following advanced clinical deterioration. Such late diagnoses are associated with poorer outcomes, including higher mortality and hospitalisation rates and delayed treatment initiation, than diagnoses made earlier in primary care. We searched PubMed from Jan 1, 2000, to Nov 1, 2024, for observational studies in English reporting diagnostic pathways in heart failure, using a combination of the following terms: “heart failure” AND “diagno∗” AND (“observational” OR “data” OR “cohort”). We included studies that reported on the application of diagnostic tests in routine practice or on the timing or location of diagnosis, and we excluded studies that focused solely on the accuracy of different heart failure diagnostic tests or novel tests. We reviewed the full text of relevant articles to assess their suitability for inclusion. Only a few studies have investigated diagnostic pathways in primary care. Most had small sample sizes and used regional data, limiting generalisability to the general population. These studies reported that more heart failure diagnoses occur in hospitals than in primary care, although findings varied, and that hospital diagnoses were associated with significantly worse mortality than primary care diagnoses. Two small studies using UK data examined diagnostic pathways. To date, no studies have investigated the association between detailed diagnostic pathways and patient outcomes.

**Added value of this study**

Our study adds value to the existing evidence by providing the largest population-level assessment of heart failure diagnosis over the past two decades. By quantifying how suboptimal and inconsistent diagnostic investigations have remained, we highlight missed opportunities for earlier diagnosis. Importantly, this study is the first to show that specific diagnostic pathways significantly influence outcomes, with patients on long-term loop diuretic therapy diagnosed in hospital without previous primary care investigations having markedly higher mortality than patients not on diuretic therapy. Finally, we identify systematic sociodemographic inequalities, highlighting patient groups that should be prioritised to reduce inequities in heart failure care.

**Implications of all the available evidence**

Taken together, the evidence shows that heart failure is often diagnosed late, with substantial variation in how and where diagnosis occurs. Our study adds robust, population-level evidence that delays and inconsistencies in diagnostic pathways are common, that they are associated with worse outcomes, and that they disproportionately affect specific patient groups. These findings suggest that improving adherence to existing guideline-recommended diagnostic approaches, ensuring timely use of confirmatory investigations, and reducing inequalities in access to care could help address gaps in early diagnosis. Further research is needed to evaluate practical strategies for earlier recognition of heart failure and to understand how system-level factors contribute to delays and variation in care.

Guidelines recommend measuring natriuretic peptides in patients with suspected heart failure, followed by specialist referral and echocardiography if concentrations are elevated.^6^^,^^7^ However, many patients ultimately diagnosed with heart failure do not follow this recommended diagnostic pathway.^1^^,^^8^ Long intervals often occur between the first recorded symptoms and a formal diagnosis, with notable disparities in care.^9^^,^^10^ These disparities persist even within universal health-care systems, driven by non-financial barriers such as factors related to patients,^11^ clinicians,^12^ and those at the system level.^13^ Although national and international guidelines over the past two decades have increasingly emphasised early diagnostic testing, it is unclear whether diagnostic practice in primary care has improved, whether patients can be identified early, before severe deterioration or hospitalisation, or how early diagnosis affects outcomes. By linking primary care, socioeconomic, hospital discharge, and mortality data, this study aimed to investigate variation in diagnostic pathways for heart failure at the population level and their association with mortality following diagnosis.

## Methods

### Study design and patients

We conducted a retrospective cohort study linking primary care data from the Clinical Practice Research Datalink (CPRD) GOLD^14^ and Aurum)^15^ databases (contributing data from practices using the Vision and EMIS Web IT systems, respectively) to the Hospital Episodes Statistics (HES) and the Office for National Statistics (ONS) death registrations. Database coverage was from Jan 1, 1985, to March 31, 2021, for primary care data and from April 1, 1997, to March 31, 2021, for secondary care data and death certificates. CPRD is a UK government-supported database containing anonymised, routinely collected electronic health records from general practices. Approximately 20% of the English population are included, providing data on patient demographics, lifestyle, consultations, clinical measurements, symptoms, diagnoses, investigations, referrals to specialist care, and medication prescriptions. HES is a government-owned national resource, managed by National Health Service (NHS) England, containing detailed information on all inpatient, outpatient, and emergency care episodes in NHS hospitals across England. At discharge, each hospital episode is assigned up to 20 International Classification of Diseases (ICD-10) codes, representing the primary diagnosis and any secondary diagnoses or relevant comorbidities.

The study protocol was approved by CPRD’s Research Data Governance Process. Ethics approval for use of CPRD data was granted by East Midlands—Derby Research Ethics Committee (21/EM/0265). The reporting of this study followed the RECORD checklist. As data were anonymised and collected during routine care by CPRD, informed consent was not required. However, patients retain the option to opt out of data sharing for research.

The eligible population included adults aged 18 years or older who were registered in CPRD, had data deemed acceptable for research (ie, met predefined CPRD quality standards for data completeness and continuity), and were from GP practices approved for linkage with HES and ONS. From this cohort, we identified all individuals with a new heart failure code in their primary care or hospital record, in any diagnostic position (ie, whether listed as the main reason for admission or as a secondary diagnosis), between Jan 1, 2000, and March 31, 2021. We excluded individuals with less than 5 years of continuous CPRD registration before diagnosis. The date of first heart failure code was defined as the diagnosis date. Diagnostic codes were compiled using WHO International Classification of Primary Care-3, CPRD, and ICD-10 browsers, cross-checked with code sets registered with HDR UK Phenotype Library and validated by clinicians.

Patient and stakeholder input shaped the study objectives and methods. At a national heart failure meeting (25in25 Summit; March 2023, London), representatives from clinical, primary care, public health, patients, and social care identified challenges in heart failure diagnosis, including sociodemographic inequalities and multiple long-term conditions. These were reiterated in two consultation groups with ten patients with a history of heart failure, who reported multiple general practitioner visits before being diagnosed, often after hospital admission. The research team included experienced Professors of Primary Care (KK and CJT) cardiologists, epidemiologists and statisticians.

### Procedures

Heart failure diagnoses were classified as outpatient (primary care, elective admission, or outpatient clinic) or inpatient (unplanned hospital admission). For hospital diagnoses, the date of admission was taken as diagnosis date. If both settings were recorded on the same day, inpatient classification was used. We identified prediagnosis indicators suggestive of heart failure, including breathlessness, ankle swelling, fatigue, or loop diuretic prescription in primary care over the preceding 5 years. Loop diuretics were included as a proxy since symptoms might not be fully coded; although occasionally used for resistant hypertension and liver or renal disease, for simplicity we assumed initiation for heart failure. Diagnostic lag time was defined as the interval between first recorded indicator and heart failure diagnosis.

We extracted natriuretic peptide (B-type natriuretic peptide [BNP] or N-terminal pro-B-type natriuretic peptide [NT-proBNP]) test results, echocardiography reports, and cardiology referrals recorded in primary care within 6 months before diagnosis and categorised patients by heart failure diagnosis location (outpatient *vs* inpatient), diagnostic investigations in primary care (≥1 investigation *vs* none), and initial presentation: (1) symptoms of heart failure without loop diuretic use before diagnosis; (2) loop diuretic use before diagnosis, with or without symptoms; and (3) no record of symptoms or loop diuretic use before diagnosis. The sequence of diagnostic steps, when recorded, is reported for each group. BNP values were converted to NT-proBNP using the formula: exp(log NT-proBNP = 1·21 + 1·03 × log BNP – 0·009 × BMI – 0·007 × eGFR),^16^ with NT-proBNP thresholds of at least 400 ng/L (NICE)^7^ and at least 125 ng/L (international guidelines).^6^ As echocardiograms in secondary care might not appear in primary care records, we considered specialist review with or without echocardiogram and reported echocardiogram separately when no review was recorded.

We linked data to the 2015 English Index of Multiple Deprivation, which measures deprivation of small neighbourhoods, ranked into quintiles (1 being the most affluent and 5 being the most deprived).^17^ Ethnicity was classified from CPRD and HES using a UK Census validated algorithm^18^ as White, south Asian, Black, mixed or other, or unknown, reflecting the most prevalent ethnicities in the 2021 census in England and Wales. South Asian included Pakistani, Indian, Bangladeshi, and other Asian ethnicities, such as Asian British, while Black included African, Caribbean, and other Black groups, such as Black British. From CPRD, we extracted age, sex (recorded as self-reported female, male, or indeterminate; individuals recorded as indeterminate were not included due to their small number), smoking status (none, ex-smoker or current smoker), blood pressure (mm Hg), cholesterol (mmol/L), BMI (kg/m^2^), haemoglobin (g/dL), and estimated glomerular filtration rate (eGFR; mL/min per m^2^; using the Chronic Kidney Disease Epidemiology Collaboration equation formula)^19^ using values closest to the time of diagnosis (before or on diagnosis date). History of 13 long-term conditions (obesity, diabetes, hypertension, chronic kidney disease, iron deficiency anaemia, atrial fibrillation, ischaemic heart disease, stroke, depression, asthma, chronic obstructive pulmonary disease, cancer, and dementia) were identified from primary or hospital records before or on the heart failure diagnosis date.

We identified prescriptions for β blockers, renin–angiotensin–aldosterone system (RAAS) inhibitors (comprising angiotensin-converting enzyme inhibitors and angiotensin II receptor blockers), angiotensin receptor neprilysin inhibitors (ARNIs), SGLT2 inhibitors, mineralocorticoid receptor antagonists (MRAs), and loop diuretics. Prescriptions were classified as: new or repeat prescriptions if prescribed 12 months to 6 months before diagnosis; newly prescribed if prescribed 0–6 months before; continued if prescribed 0–6 months after diagnosis; newly prescribed if prescribed 0–6 months after; and discontinued if discontinued within 6 months after diagnosis.

### Outcomes

The primary outcome was deaths within 1 year of diagnosis. The secondary outcome was deaths within 5 years of diagnosis. The date of death was obtained from linked ONS records in all patients.

### Statistical analysis

We described patient characteristics at heart failure diagnosis stratified by diagnosis setting. We examined early indicators (symptoms and loop diuretic use) and diagnostic investigations in primary care. For descriptive tables, we compared two time periods, 2000–04 and 2015–19. These time windows were chosen to contrast the period before (2000–04) versus after (2015–19) the introduction of national guidance promoting earlier diagnosis and management of heart failure. Although natriuretic peptide testing for heart failure was more firmly established in the 2010 NICE guidelines, the role of natriuretic peptide was mentioned in the 2003 guidelines in terms of its potential value in diagnosis. To assess the effect of COVID-19 restrictions on primary care services, 2019–20 was also compared. To assess more detailed trends among patients with previous indicators, logistic regression estimated age-adjusted odds of natriuretic peptide testing, echocardiography, and specialist referral per year (2000–20), with year–age interaction. Probabilities were estimated using predicted margins^20^ at mean age 78 years and plotted. Joinpoint regression estimated annual percentage change and significance of temporal trend (appendix p 3).

Given the evolving diagnostic coding and use of diagnostic investigations, all further analyses (including diagnostic pathways, guideline-recommended medical therapy [GRMT] prescriptions, sociodemographic group comparisons, and survival) were restricted to patients diagnosed with heart failure between Jan 1, 2015, and Dec 31, 2019. We described diagnostic pathways for three groups: symptoms only, loop diuretic initiation (with or without symptoms), and neither and analyses were stratified by diagnosis setting. To assess sociodemographic variation among patients with previous indicators, logistic regression compared: (1) women versus men; (2) most deprived versus most affluent neighbourhoods; (3) south Asian and Black versus White; and (4) four or more versus fewer than four long-term conditions (multiple long-term conditions, median split). Models were adjusted for age, sex, ethnicity, deprivation, year, systolic blood pressure, BMI, cholesterol, smoking, and comorbidities (except in the multiple long-term condition model). Linearity of continuous covariates was assessed with likelihood ratio tests, and p values <0.05 were considered evidence of non-linearity.

We examined survival using Royston–Parmar flexible parametric survival models adjusting for the same covariates and therapies. Given low missingness in confounders and unlikely association with mortality once conditioned, we used complete case analysis.^21^ Hazard ratios (HRs) and 95% CIs were calculated. Patients were followed from diagnosis until death or March 31, 2021; survival was predicted at 1 year and 5 years. All analyses were conducted in Stata MP, version 18.

We conducted three sensitivity analyses. First, we assessed heart failure case identification robustness (appendix pp 4–6). Briefly, using a published approach,^22^ we compared 5-year survival in three heart failure coding groups: (1) primary care only, (2) both primary and secondary care, and (3) secondary care only. Next, we refined the primary care only group into code only without diagnostic test or GRMT and code only with at least one diagnostic test or GRMT. Finally, we repeated the 1-year mortality models, excluding the patients with only a primary care code and no diagnostic test or GRMT. For sociodemographic group comparisons for the second sensitivity analysis, we used multilevel mixed-effects logistic regression with general practice as a random effect to estimate intraclass correlation coefficients (ICC), which quantified variation attributable to practice-level differences, and adjusted for this in the analysis. For the third sensitivity analysis, we included patients who died on their heart failure index date for all analyses because our goal was to examine how diagnosis pathways relate to outcomes. Death or hospital diagnosis on the index date are important outcomes when assessing diagnostic delays; therefore, we conducted a post-hoc sensitivity analysis, excluding these patients from the survival models.

### Role of the funding source

The funders of the study had no role in study design, data collection, data analysis, data interpretation, or writing of the report.

## Results

From 10 449 677 adults in 1662 general practices, 412 173 had a new heart failure diagnosis between Jan 1, 2000, and March 31, 2021 (appendix p 7), with median follow-up of 4·9 years (IQR 1·5–10·6). Of 412 173 individuals, 230 886 (56·0%) were diagnosed in outpatient and 181 287 (44·0%) in inpatient settings, the median age at diagnosis was 78·0 years (IQR 69·0–85·0), 217 998 (52·9%) were men, and 194 175 (47·1%) were women (table 1). In 407 622 participants with ethnicity data, ethnicity was recorded as White for 375 808 (92·2%), south Asian for 11 644 (2·9%), Black for 6994 (1·7%), other or mixed for 3622 (0·9%), and unknown for 9554 (2·3%). Compared with outpatients, inpatients were older, more often women, from deprived quintiles, and had more comorbidities.

**Table 1:** Patients characteristics at time of heart failure diagnosis by location of diagnosis

	Outpatient (n=230 886)	Inpatient (n=181 287)	Total (n=412 173)
Age at diagnosis, years	77·0 (68·0–83·0)	79·0 (70·0–86·0)	78·0 (69·0–85·0)
Sex			
Male	127 595 (55·3%)	90 403 (49·9%)	217 998 (52·9%)
Female	103 291 (44·7%)	90 884 (50·1%)	194 175 (47·1%)
Ethnicity			
White	209 405/226 777 (92·3%)	166 403/180 845 (92·0%)	375 808/407 622 (92·2%)
South Asian	6137/226 777 (2·7%)	5507/180 845 (3·0%)	11 644/407 622 (2·9%)
Black	3730/226 777 (1·6%)	3264/180 845 (1·8%)	6994/407 622 (1·7%)
Other or mixed	2014/226 777 (0·9%)	1608/180 845 (0·9%)	3622/407 622 (0·9%)
Unknown	5491/226 777 (2·4%)	4063/180 845 (2·2%)	9554/407 622 (2·3%)
Index of Multiple Deprivation level			
1 (most affluent)	46 922/230 354 (20·4%)	32 468/180 649 (18·0%)	79 390/411 003 (19·3%)
2	49 163/230 354 (21·3%)	35 779/180 649 (19·8%)	84 942/411 003 (20·7%)
3	46 195/230 354 (20·1%)	35 757/180 649 (19·8%)	81 952/411 003 (19·9%)
4	44 808/230 354 (19·5%)	37 300/180 649 (20·6%)	82 108/411 003 (20·0%)
5 (most deprived)	43 266/230 354 (18·8%)	39 345/180 649 (21·8%)	82 611/411 003 (20·1%)
Smoking status			
Non-smoker	100 003/219 246 (45·6%)	78 602/173 248 (45·4%)	178 605/392 494 (45·5%)
Ex-smoker	73 495/219 246 (33·5%)	56 071/173 248 (32·4%)	129 566/392 494 (33·0%)
Current smoker	45 748/219 246 (20·9%)	38 575/173 248 (22·3%)	84 323/392 494 (21·5%)
Comorbidities			
Comorbidity count	3·0 (2·0–5·0)	4·0 (3·0–5·0)	4·0 (2·0–5·0)
Ischaemic heart disease	104 789 (45·4%)	93 883 (51·8%)	198 672 (48·2%)
Myocardial infarction	79 922 (34·6%)	80 350 (44·3%)	160 272 (38·9%)
Hypertension	154 713 (67·0%)	134 062 (74·0%)	288 775 (70·1%)
Atrial fibrillation	77 441 (33·5%)	83 973 (46·3%)	161 414 (39·2%)
Obesity	66 787 (28·9%)	52 971 (29·2%)	119 758 (29·1%)
Diabetes	55 880 (24·2%)	55 421 (30·6%)	111 301 (27·0%)
Chronic kidney disease (coded or eGFR <60 mL/min per m^2^)	84 784 (36·7%)	77 520 (42·8%)	162 304 (39·4%)
Iron deficiency anaemia	24 511 (10·6%)	29 203 (16·1%)	53 714 (13·0%)
Stroke	24 244 (10·5%)	26 132 (14·4%)	50 376 (12·2%)
Asthma	47 307 (20·5%)	41 017 (22·6%)	88 324 (21·4%)
Chronic obstructive pulmonary disease	39 420 (17·1%)	42 593 (23·5%)	82 013 (19·9%)
Cancer	60 059 (26·0%)	51 262 (28·3%)	111 321 (27·0%)
Depression	10 988 (4·8%)	13 606 (7·5%)	24 594 (6·0%)
Dementia	8895 (3·9%)	13 140 (7·2%)	22 035 (5·3%)
Clinical measures			
eGFR (mL/min per m^2^)	63 (49–79), n=193 208	60 (45–77), n=154 550	62 (47–78), n=347 758
Systolic blood pressure (mm Hg)	136 (124–148), n=225 837	135 (121–146), n=176 881	136 (122–147), n=402 718
Diastolic blood pressure (mm Hg)	78 (70–83), n=224 120	77 (70–82), n=175 485	78 (70–83), n=399 605
Haemoglobin (g/dL)	13 (12–15), n=194 587	13 (12–14), n=156 753	13 (12–14), n=351 340
BMI (kg/m^2^)	28 (24–32), n=201 086	27 (234–32), n=158 414	28 (24–32), n=359 500
Cholesterol (mmol/L)	5 (4–5), n=181 725	5 (4–5), n=145 178	5 (4–5), n=326 903
Drug prescriptions			
β blocker	55 181 (23·9%)	35 779 (19·7%)	90 960 (22·1%)
Angiotensin-converting enzyme inhibitor	88 601 (38·4%)	59 609 (32·9%)	148 210 (36·0%)
Angiotensin II receptor blockers	31 771 (13·8%)	24 346 (13·4%)	56 117 (13·6%)
Mineralocorticoid receptor antagonist	10 843 (4·7%)	7686 (4·2%)	18 529 (4·5%)
Angiotensin receptor neprilysin inhibitor	72 (<0·1%)	20 (<0·1%)	92 (<0·1%)
SGLT2 inhibitor	663 (0·3%)	569 (0·3%)	1232 (0·3%)
Loop diuretic	88 683 (38·4%)	67 459 (37·2%)	156 142 (37·9%)

Data are median (IQR), n (%), or n/N (%). Sample size is shown if there were missing data. eGFR=estimated glomerular filtration rate. Prescribed drugs were identified by at least one prescription in a 6-month time window before heart failure diagnosis.

Overall, 196 514 (47·7%) of 412 173 individuals with a new heart failure diagnosis presented with symptoms a median 23·4 months (IQR 3·6–46·2) before diagnosis, and 187 683 (45·5%) of 412 173 patients received loop diuretics 17·3 months (1·6–52·9) before diagnosis. The median time from the first of these indicators was 28·5 months (3·9–52·9). The most common symptom was shortness of breath (161 238 [39·1%] of 412 173), followed by fatigue (40 894 [9·9%] of 412 173), and ankle swelling (40 558 [9·8%] of 412 173). Lag times were longer for inpatient than outpatient diagnoses, especially among people given loop diuretics (30·0 months [5·0–58·3] *vs* 9·0 months [0·9–45·1]; table 2). Over time, the recording of heart failure symptoms in primary care increased from 29 294 (32·5%) of 90 136 patients in 2000–04 to 64 028 (53·6%) of 119 355 patients in 2015–19. In parallel, diagnostic lag times increased for individuals with previous symptoms (from a median of 10·0 months [1·2–30·5] to 31·1 months [5·9–50·5]). Diagnostic lag times for individuals with previous loop diuretic use was stable for patients diagnosed in outpatient settings but increased from a median of 24·7 (4·0–55·4) to 31·7 months (5·8–58·6) for patients diagnosed in inpatient settings (table 2).

**Table 2:** Diagnosis location, previous presentation in primary care, and diagnostic processes recorded in primary care in the 6 months before heart failure diagnosis

	2000–04 (n=90 136)	2015–19 (n=119 355)	All (n=412 173)
**Location of heart failure diagnosis**			
Outpatient setting	59 576 (66·1%)	63 450 (53·2%)	230 886 (56·0%)
Inpatient setting	30 560 (33·9%)	55 905 (46·8%)	181 287 (44·0%)
**Presentation in primary care with features suspicious of heart failure**			
Any symptom or loop diuretic use			
Yes	55 812 (61·9%)	80 824 (67·7%)	274 228 (66·5%)
No	34 324 (38·1%)	38 531 (32·3%)	137 945 (33·5%)
Any heart failure symptom consultation	29 294 (32·5%)	64 028 (53·6%)	196 514 (47·7%)
Shortness of breath	23 799 (26·4%)	52 248 (43·8%)	161 238 (39·1%)
Fatigue	5687 (6·3%)	13 581 (11·4%)	40 894 (9·9%)
Ankle swelling	4980 (5·5%)	14 300 (12·0%)	40 558 (9·8%)
Symptom only	11 757 (13·0%)	30 222 (25·3%)	86 545 (21·0%)
Loop diuretic only	26 518 (29·4%)	16 796 (14·1%)	77 714 (18·9%)
Loop diuretic with or without symptom	44 055 (48·9%)	50 602 (42·4%)	187 683 (45·5%)
**Time from first features suspicious of heart failure to diagnosis, months**			
Outpatient diagnosis			
Time from first heart failure symptom to diagnosis	10·5 (1·5–31·1)	28·8 (5·4–49·6)	21·8 (3·6–44·9)
Time from first loop diuretic to diagnosis	8·7 (0·4–42·7)	8·9 (1·1–45·2)	9·0 (0·9–45·1)
Time from first indicator to diagnosis	14·5 (1·3–44·0)	30·4 (4·3–52·8)	23·9 (3·0–50·5)
Inpatient diagnosis			
Time from first heart failure symptom to diagnosis	9·0 (0·5–29·4)	33·6 (6·9–51·3)	25·7 (3·6–47·6)
Time from first loop diuretic to diagnosis	24·7 (4·0–55·4)	31·7 (5·8–58·6)	30·0 (5·0–58·3)
Time from first indicator to diagnosis	21·0 (2·6–49·1)	40·8 (10·6–56·5)	34·3 (7·0–55·4)
All			
Time from first heart failure symptom to diagnosis	10·0 (1·2–30·5)	31·1 (5·9–50·5)	23·4 (3·6–46·2)
Time from first loop diuretic to diagnosis	12·9 (0·9–46·9)	19·5 (1·8–54·3)	17·3 (1·6–52·9)
Time from first indicator to diagnosis	16·4 (1·6–45·7)	35·4 (6·4–54·7)	28·5 (3·9–52·9)
**Diagnostic pathway elements by previous indicators of heart failure**			
With recorded symptoms or loop diuretic use			
At least one natriuretic peptide test	NA	10 079/80 824 (12·5%)	21 909/274 228 (8·0%)
Highest NT-proBNP value			
<125 ng/L	NA	634/8772 (7·2%)	1316/19 363 (6·8%)
125–400 ng/L	NA	1498/8772 (17·1%)	3288/19 363 (17·0%)
≥400 ng/L	NA	6640/8772 (75·7%)	14 759/19 363 (76·2%)
Missing	NA	1307/10 079 (13.0%)	2546/21 909 (11·6%)
Echocardiogram	3614/55 812 (6·5%)	15 986/80 824 (19·8%)	43 723/274 228 (15·9%)
At least one cardiology specialist review	4049/55 812 (7·3%)	27 804/80 824 (34·4%)	67 325/274 228 (24·6%)
Any pathway element starting in primary care	6953/55 812 (12·5%)	38 522/80 824 (47·7%)	99 187/274 228 (36·2%)
No pathway element starting in primary care	48 859/55 812 (87·5%)	42 302/80 824 (52·3%)	175 041/274 228 (63·8%)
Without recorded symptoms or loop diuretic use			
At least one natriuretic peptide test	NA	1419/38 531 (3·7%)	2869/137 945 (2·1%)
Highest NT-proBNP value			
<125 ng/L	NA	142/1237 (11·5%)	248/2542 (9·8%)
125–400 ng/L	NA	227/1237 (18·4%)	500/2542 (19·7%)
≥400 ng/L	NA	868/1237 (70·2%)	1794/2542 (70·6%)
Missing		182/1419 (12·8%)	327/2869 (11·4%)
Echocardiogram	2350/34 324 (6·8%)	7798/38 531 (20·2%)	20 964/137 945 (15·2%)
At least one cardiology specialist review	1923/34 324 (5·6%)	11 309/38 531 (29·4%)	26 771/137 945 (19·4%)
Any pathway element starting in primary care	3852/34 324 (11·2%)	15 807/38 531 (41·0%)	40 467/137 945 (29·3%)
No pathway element starting in primary care	30 472/34 324 (88·8%)	22 724/38 531 (59·0%)	97 478/137 945 (70·7%)

Data are n (%), median (IQR), or n/N (%). Any pathway: at least one of natriuretic peptide test, echocardiogram, or specialist review. No pathway: none of natriuretic peptide test, echocardiogram, or specialist review. NA indicates statistics not reported due to low numbers. NA=not applicable. NT-proBNP=N-terminal pro-B-type natriuretic peptide.

**Figure 1:**
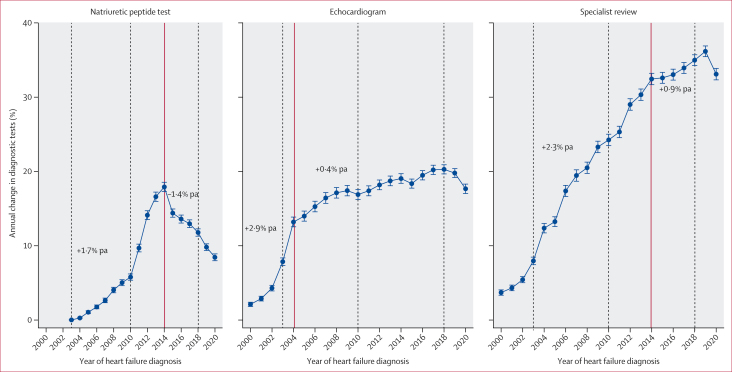
Annual percentage change and 95% CI in diagnostic tests and specialist review over time Trends in diagnostic processes recorded in primary care in the 6 months before diagnosis by calendar year of diagnosis. Error bars show 95% CI. Models were restricted to patients with a previous indication of heart failure (symptom or loop diuretic use; n=274 228). The red solid line indicates a statistically significant change point, representing a change in trend from the preceding time segment identified by joinpoint regression (appendix p 3). Introduction of different UK National Institute for Health and Care Excellence guidelines for heart failure are denoted by the dark grey dotted lines at 2003, 2010, and 2018. pa=per annum.

Among 274 228 (66·5%) of 412 173 patients with previous indicators suggestive of heart failure, rates of investigations recorded in primary care were low and improved only modestly over time (table 2). Of 80 824 individuals with previous indicators in 2015–19, 10 079 (12·5%) had natriuretic peptide testing, 27 804 (34·4%) were referred for specialist review, 15 986 (19·8%) had echocardiography recorded, and 42 302 (52·3%) had no primary care record of confirmatory tests. Natriuretic peptide testing (+1·7% per annum [pa]) and specialist referral (+2·3% pa) rose until 2014, after which natriuretic peptide testing declined (–1·4% pa) and specialist referral slowed (+0·9% pa) (figure 1). Echocardiographic recording increased steeply until 2004 (+2·9% pa), then plateaued (+0·4% pa). Diagnostic activity fell at the start of COVID-19 in 2020 versus 2019 (figure 1; appendix p 8). Inpatient diagnoses rose from 3017 (26·4%) of 11 425 patients in 2001 to 6018 (48·0%) of 12 545 patients in 2011 (+2·7% pa) before plateauing, then surged to 7295 (51·8%) of 14 085 patients in 2020 (+4·4%; appendix p 9). Trends were similar in the 137 945 (33·5%) of 412 173 patients without previous indicators but natriuretic peptide testing was rare (1419 [3·7%] of 38 531 patients in 2015–19; table 2).

Given the variation in diagnostic investigations over time, the following analyses were restricted to the most recent pre-COVID-19 years (ie, Jan 1, 2015, to Dec 31, 2019). Among 42 830 outpatients with previous indicators, 28 996 (67·7%) had at least one investigation in the 6 months before diagnosis. Investigation rates were similar among patients with or without loop diuretic prescription, although lag times were shorter after diuretic initiation, particularly for patients who had diagnostic investigations initiated (appendix p 10). Specialist referral with or without echocardiography (approximately 40%) was most common; natriuretic peptide pathways were least used. Investigations were far less common in inpatients with previous indicators (2777 [18·8%] of 14 787 patients with previous symptoms and 6749 [29·1%] of 23 207 patients following loop diuretic initiation; appendix p 11). In those without investigations, median time to inpatient diagnosis was 30·7 months (IQR 3·2–50·5) from symptoms and 37·1 months (10·0–59·0) from diuretic initiation. Patients without recorded symptoms or diuretic use had broadly similar pathways (appendix p 12).

Of 119 355 individuals diagnosed with heart failure between 2015 and 2019, 76 010 (63·7%) received GRMT before diagnosis (appendix pp 14–16), mainly RAAS inhibitors (60 804 [50·9%]) and β blockers (40 005 [33·5%]). Regardless of diagnosis location (inpatient or outpatient), new GRMT prescribing in the 6 months before diagnosis was higher for individuals who had diagnostic processes initiated in primary care than in those who had no diagnostic processes recorded (outpatients 5779 [13·6%] of 42 426 *vs* 1364 [6·5%] of 21 014; inpatients 1399 [11·8%] of 11 903 *vs* 1621 [3·7%] of 44 002). Of 113 665 individuals alive 1 month after diagnosis, prescribing rose by approximately 17%, with 91 572 (80·6%) on at least one GRMT (RAAS 74 332 [65·4%%]; β blocker 65 935 [58·0%]). MRA use was rare before diagnosis (5708 [4·8%] of 119 355 patients) and modest after diagnosis (23 390 [20·6%] of 113 665 patients). Given their recommendation in guideline updates (2016 for ARNIs^23^^,^^24^ and 2021 for SGLT2 inhibitors^6^^,^^25^), these therapies were rarely prescribed during the 2015–19 study period. Prescriptions were highest in those on loop diuretics and modestly higher in those with symptoms compared with those with no previous indicators recorded.

Symptoms or loop diuretic use were more often recorded in women (136 383 [70·2%] of 194 175) than in men (137 845 [63·2%] of 217 998), in individuals who were White (251 857 [67·0%] of 375 808) than in individuals who were south Asian (7264 [62%] of 11 644) or Black (4206 [60·1%] of 6994), and in those with four or more multiple long-term conditions (159 184 [74·3%] of 214 288) than those with fewer than four (115 044 [58·1%] of 197 885), but there was little difference by deprivation (appendix p 17). During 2015–19, lag times showed marked disparities: among individuals on loop diuretics (n=50 602), women waited about twice as long as men (25·9 months [IQR 2·6–57·6] *vs* 13·3 [IQR 1·4–48·6] months) and individuals living in the most deprived quintile waited 8 months longer than individuals in the most affluent (23·7 months [IQR 2·5–57·5] *vs* 16·2 months [IQR 1·5–50·9]). Patients with at least four multiple long-term conditions waited almost five times longer than those with fewer than four (27·0 months [IQR 3·3–57·7] *vs* 4·7 months [IQR 0·8–36·5]; appendix p 18). Following adjustment, of those with previous indicators, women compared with men (odds ratio 0·86 [95% CI 0·83–0·89]), individuals living in the most deprived compared with the most affluent quintile (0·82 [0·78–0·86]), and those with at least four multiple long-term conditions compared with those with less (0·78 [0·76–0·81]) were significantly less likely to undergo any diagnostic investigations. Women (1·20 [1·17–1·24]), individuals living in the most deprived quintile (1·29 [1·23–1·36]), and those with at least four multiple long-term conditions (1·89 [1·83–1·95]) were also more likely to be diagnosed in inpatient settings than men, the most affluent, and individuals with fewer conditions, respectively. Ethnicity differences were also apparent, although with more nuanced variations. Compared with White individuals, south Asian and Black individuals were less likely to receive natriuretic peptide testing or echocardiography, and south Asians were more likely to receive their diagnosis in hospital (appendix pp 20–22).

**Figure 2:**
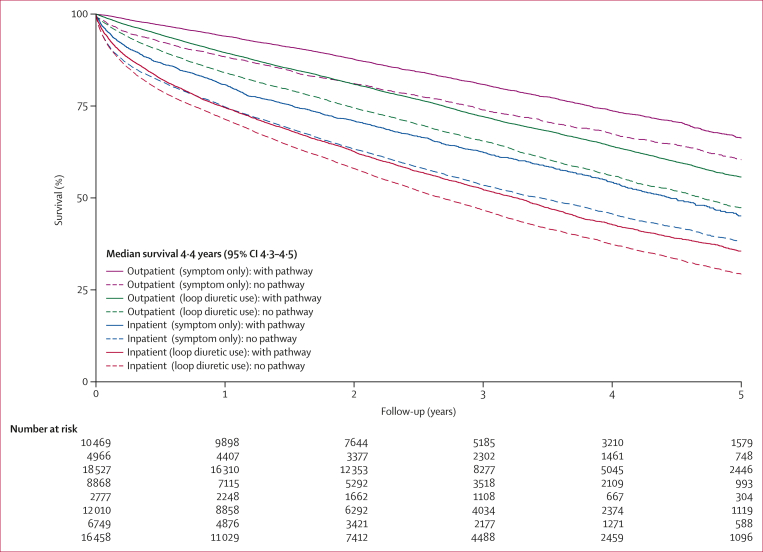
Kaplan–Meier plot of age-adjusted survival following heart failure diagnosis by diagnosis group Survival predicted at the mean population age (78 years). Solid lines are groups with primary care-initiated processes before diagnosis. Dashed lines are groups without primary care-initiated processes before diagnosis.

Waiting more than 6 months for diagnosis after first loop diuretic prescription was linked to higher 1-year mortality compared with shorter wait times (adjusted HR 1·15 [95% CI 1·10–1·20]). Absence of primary care diagnostic investigations (1·89 [1·83–1·95]) and inpatient diagnosis (2·58 [2·50–2·66]) were also associated with excess risk. Among patients with indicators (eg, symptoms or loop diuretic use) patients with diagnostic actions initiated in primary care had better survival (figure 2, solid lines) than those without recorded diagnostic actions (figure 2, dashed lines), regardless of diagnosis setting. 1-year mortality was lowest for outpatients with primary care investigations (5·5% in patients with previous symptom only), but higher for patients using loop diuretics, those without diagnostic testing initiated in primary care, and those diagnosed during hospitalisation (reaching 33·0% in patients with these issues combined; adjusted HR 5·29 [95% CI 4·83–5·79]; table 3). Mortality at 5 years was higher but with a similar pattern of variations among the patient groups. Survival was worse in men than women, although diagnostic-pathway effects were similar across sexes (appendix pp 23–24).

**Table 3:** Associations between diagnostic pathways and mortality for patients with heart failure according to location of diagnosis and whether symptoms only or loop diuretic initiation preceded the diagnosis for 2015–19

	Mortality	Unadjusted HR (95% CI)	Adjusted∗ HR (95% CI)
**1-year mortality rate**			
Outpatient (previous symptom only): with pathway	571/10 469 (5.5%)	1 (ref)	1 (ref)
Outpatient (previous symptom only): no pathway	559/4966 (11·3%)	2·14 (1·91–2·41)	1·91 (1·69–2·15)
Outpatient (previous loop diuretic use): with pathway	2217/18 527 (12·0%)	2·26 (2·06–2·48)	2·01 (1·83–2·21)
Outpatient (previous loop diuretic use): no pathway	1753/8868 (19·8%)	3·93 (3·57–4·32)	3·11 (2·82–3·43)
Inpatient (previous symptom only): with pathway	529/2777 (19·0%)	3·81 (3·38–4·28)	3·46 (3·07–3·92)
Inpatient (previous symptom only): no pathway	3152/12 010 (26·2%)	5·55 (5·08–6·07)	4·43 (4·04–4·86)
Inpatient (previous loop diuretic use): with pathway	1873/6749 (27·8%)	5·85 (5·33–6·42)	4·87 (4·41–5·37)
Inpatient (previous loop diuretic use): no pathway	5429/16 458 (33·0%)	7·28 (6·68–7·94)	5·29 (4·83–5·79)
**5-year mortality rate**			
Outpatient (previous symptom only): with pathway	2082/10 469 (19·9%)	1 (ref)	1 (ref)
Outpatient (previous symptom only): no pathway	1352/4966 (27·2%)	1·44 (1·35–1·54)	1·31 (1·22–1·41)
Outpatient (previous loop diuretic use): with pathway	6439/18 527 (34·8%)	1·87 (1·78–1·97)	1·61 (1·53–1·69)
Outpatient (previous loop diuretic use): no pathway	4048/8868 (45·6%)	2·69 (2·55–2·83)	2·11 (2·00–2·23)
Inpatient (previous symptom only): with pathway	1115/2777 (40·2%)	2·37 (2·20–2·55)	2·14 (1·99–2·31)
Inpatient (previous symptom only): no pathway	5931/12 010 (49·4%)	3·23 (3·07–3·40)	2·62 (2·48–2·75)
Inpatient (previous loop diuretic use): with pathway	3654/6749 (54·1%)	3·61 (3·42–3·80)	2·94 (2·78–3·11)
Inpatient (previous loop diuretic use): no pathway	10 261/16 458 (62·3%)	4·56 (4·35–4·78)	3·23 (3·07–3·39)

Data are n/N (%), unless otherwise stated. Any pathway: at least one of natriuretic peptide test, echocardiogram, or specialist review, recorded in the primary care record in the 6 months before heart failure diagnosis. No pathway: none of natriuretic peptide test, echocardiogram, or specialist review, recorded in the primary care record in the 6 months before heart failure diagnosis. Models were restricted to patients with a previous indication of heart failure (symptom or loop diuretic use) and diagnosed with heart failure before COVID-19 (2015**–**19; n=80 824). HR=hazard ratio.

∗ Adjusted models (n=75 927) included age, sex, ethnicity, socioeconomic status, year of diagnosis, systolic blood pressure, BMI, cholesterol, smoking, comorbidities (ie, ischaemic heart disease, hypertension, atrial fibrillation, diabetes, chronic kidney disease, iron deficiency anaemia, stroke, asthma, chronic obstructive pulmonary disease, cancer, depression, and dementia), and prescribed drugs at diagnosis (ie, β blocker, renin–angiotensin–aldosterone system inhibitors, mineralocorticoid receptor antagonists, angiotensin receptor neprilysin inhibitors, and SGLT2 inhibitors).

In the sensitivity analyses, excluding individuals with only a primary care code and no diagnostic test or GRMT prescription did not alter associations (appendix p 25). Practice-level variation was greatest for natriuretic peptide testing (ICC 0·51) and echocardiography (ICC 0·74; appendix p 26). After adjusting for practice variation, the difference in natriuretic peptide testing between socioeconomic groups and in diagnostic investigations by ethnicity lost statistical significance. However, disparities by sex, deprivation, and multimorbidity remained. Place of diagnosis was unaffected by practice variation. Patients who died on their index date were slightly older and more likely to be in the most deprived quintile. They were also more likely to be diagnosed in the outpatient setting and less likely to be prescribed GRMTs before their diagnosis (appendix p 27). Removing patients who died on their index date from the survival models had little impact on the associations with mortality (appendix p 28).

## Discussion

In this nationally representative sample of patients with newly diagnosed heart failure over the past 20 years in England, diagnostic investigations initiated in primary care were below guideline standards, with little evidence of improvement in the past decade. Between 2015 and 2019, 46·8% of heart failure diagnoses were first made during inpatient stays, despite 66·5% of patients presenting with suggestive symptoms or being prescribed loop diuretics an average of 2·5 years before diagnosis. Among those with prior indicators, natriuretic peptide testing, a key recommended first-line investigation, was requested in primary care for only 12·5% of patients. Direct referral for specialist review (34·4%) was the most common diagnostic pathway, and 52·3% of patients had no primary care record of confirmatory tests. Patients first diagnosed in hospital had a 1-year mortality 4–5 times higher than outpatients, particularly after long-term loop diuretic use and when no previous diagnostic actions were initiated in primary care. Delays in diagnosis probably lead to prolonged congestion, raising risks of complications, hospitalisation, and death. Disparities in care, which have substantial consequences, were evident. Women and individuals with socioeconomic deprivation and multimorbidity were less likely to undergo diagnostic investigations and more often diagnosed during emergency admissions. Patients without diagnostic actions initiated in primary care were less likely to receive new GRMT prescribed before diagnosis and had a poorer survival following diagnosis than individuals with diagnostic actions.

Earlier CPRD data (2010–13) suggested 79% of heart failure diagnoses occurred in hospital,^1^ a figure widely cited. However, our larger, more contemporary cohort indicates a substantially lower figure. This discrepancy might result from general practice data gaps in earlier studies. Our inclusion of patients with 5 years of continuous data and classification of elective admissions as outpatient diagnoses yielded hospital diagnosis rates of approximately 50%, consistent with previous CPRD analyses (2007–10),^16^ using a similar approach. Diagnostic delays from first symptom presentation are largely unchanged,^9^ showing little improvement in recent years.

Natriuretic peptide testing is sensitive, cost-effective,^26^ and widely available but is underused in UK primary care, with inappropriate and repeat testing^27^ suggesting a need for improved education and support for overburdened general practitioners. Echocardiography, although widely performed nationally,^28^ shows major variation in primary care access, with most tests tied to specialist review, often involving long waiting lists^29^ and specialists might not provide a detailed echocardiographic report. Broad natriuretic peptide testing could act as a gateway, reducing unnecessary referrals and expediting evidence-based therapy. However, as our analysis only includes patients eventually diagnosed with heart failure, we cannot comment on how many referrals might have been prevented by natriuretic peptide testing.

Women and individuals living in deprived quintiles were less likely to see specialists, consistent with earlier work.^30^ Older age, comorbidities, and differing risk profiles^31^ probably contribute to these care gaps, highlighting the need for collaborative efforts between policy makers, clinicians, and health services to improve equitable access. For patients with frailty or those with multimorbidity, intensive specialist investigation might not be appropriate, and community-based pathways, such as early screening, remote specialist input, and local management,^32^ might be more suitable and cost-effective.^33^

A key finding was the high rate of loop diuretic initiation years before diagnosis, especially in patients later diagnosed during hospitalisation. Loop diuretics are often prescribed to manage symptoms and signs of congestion and even in the absence of a diagnosis of heart failure, are associated with a poor prognosis.^3^ Although some patients might be treated with loop diuretics for resistant hypertension or end-stage kidney disease, many of these patients are likely to have undiagnosed or subclinical heart failure.^3^^,^^4^ More patients are prescribed loop diuretics than get a diagnosis of heart failure but mortality rates of those treated with loop diuretics are similar whether or not they are diagnosed with heart failure.^4^ As most patients started on loop diuretics die without a heart failure diagnosis, the true scale of missed or delayed diagnosis could be worse than our data suggest.

By linking large datasets across two decades, we provide a detailed picture of diagnostic trends and inequalities in heart failure care. Our findings extend previous work by using contemporary data in a large cohort and linking diagnostic pathways with outcomes, with a detailed analysis of sociodemographic and clinical inequalities. Use of routinely collected clinical data means misclassification of heart failure cannot be ruled out, particularly in outpatient cases without confirmatory tests. However, diagnostic precision in CPRD is generally good,^16^ and diagnostic testing was strongly linked with reduced risk. We acknowledge that coding accuracy can vary, so we conducted sensitivity analyses comparing survival by source of diagnosis; results mirrored those of previous work,^16^ showing that even when heart failure was coded only in primary care, mortality remained substantially elevated compared with the general population and similar to hospital coded heart failure, suggesting that such codes identify clinically important disease. The generalisability of our analyses is limited by the under-representation of some ethnicities and unavailability of specific variables relevant to the characterisation of heart failure phenotypes, such as values of ejection fraction. Furthermore, diagnostic investigations performed during hospital admissions were not available in the studied dataset and might only be partly documented in patients’ primary care records. Many patients treated with loop diuretics did not have heart failure symptoms recorded, suggesting under-recording of symptoms. Furthermore, the non-specific nature of heart failure symptoms and the varied clinical indications for loop diuretics limit the ability to determine diagnostic timing with complete accuracy. Nonetheless, in the context of routine primary care data, proxies, such as loop diuretic prescriptions, and symptoms, such as breathlessness, represent the best available indicators of early clinical manifestations of heart failure. We recognise that delays in referring patients for natriuretic peptide testing and specialist examination, including echocardiography, might have been influenced by administrative limitations, such as referral protocols, regional access to diagnostic services, and waiting times for specialist assessments, although these factors could not be directly assessed in this study due to the limitations of our dataset.

In conclusion, diagnostic testing and specialist review for heart failure is suboptimal, particularly among women, individuals living in deprived quintiles, specific ethnicities, and those with multimorbidity. Failure to investigate promptly delays GRMT initiation, increases hospital-based diagnoses, and worsens prognosis. These findings show that guideline-recommended care is not being consistently followed, highlighting the need for evidence-based strategies to improve heart failure diagnosis and management. Potential measures could include promoting routine natriuretic peptide testing in patients with suggestive symptoms or those initiated on loop diuretics without a clear alternative indication, expanding access to specialist review through telemedicine or community diagnostic hubs, and embedding digital prompts and decision-support tools within primary care systems, although the effectiveness of these approaches require further evaluation. Improved understanding of patient presentation patterns, barriers to care, and system-level contributors to delays and inequities is also needed. A coordinated effort involving policy makers, clinicians, and health-care services will be crucial to translate guidelines into practice, reduce disparities, and improve outcomes for patients with heart failure.

## Data sharing

Data are available from the Clinical Practice Research Datalink (CPRD) and Office for National Statistics (ONS) directly. A detailed protocol can be provided on request to CAL. Code lists are available on GitHub at https://github.com/CAL-CVS/THINK-HF.git. CPRD primary care data: this study is based on data from the CPRD obtained under licence from the UK Medicines and Healthcare Products Regulatory Agency. The data are provided by patients and collected by the UK National Health Service as part of their care and support. Hospital Episode Statistics and ONS data: re-used with the permission of The Health & Social Care Information Centre. All rights reserved. The interpretation and conclusions contained in this study are those of the authors alone.

## Declaration of interests

CAL has acted as a speaker for Boehringer Ingelheim. KK has acted as a consultant, speaker, or received grants for investigator-initiated studies for AstraZeneca, Boehringer Ingelheim, Lilly, MSD, Novo Nordisk, Sanofi, Servier, Oramed Pharmaceuticals, Roche, Daiichi-Sankyo, and Applied Therapeutics; received consulting fees from Amgen, AstraZeneca, Bristol Myers Squibb, Boehringer Ingelheim, Lilly, Novo Nordisk, Sanofi, Servier, Pfizer, Roche, Daiichi-Sankyo, Embecta, and Nestlé Health Science; and received payment or honoraria from Amgen, AstraZeneca, Bristol Myers Squibb, Boehringer Ingelheim, Lilly, Novo Nordisk, Sanofi, Servier, Pfizer, Roche, Daiichi-Sankyo, Embecta, and Nestlé Health Science. FZ has acted as a consultant or speaker for Servier, Menarini, Daiichi-Sankyo. CAM has received grants or contracts from National Institute for Health and Care Research (NIHR), British Heart Foundation (BHF), and Roche; participated on advisory boards or consulted for AstraZeneca, Boehringer Ingelheim and Lilly Alliance, Novartis, and PureTech Health; serves as an advisor for HAYA Therapeutics; has received speaker fees from AstraZeneca, Boehringer Ingelheim, and Novo Nordisk; conference attendance support from AstraZeneca; and research support from Amicus Therapeutics, AstraZeneca, Guerbet Laboratories, Roche, and Univar Solutions. NC has received grants from Wellcome Trust, Research Foundation Flanders, and KU Leuven. JMF has received grants from the Scottish Government and Greater Glasgow & Clyde National Health Service. CJT has received grants from the BHF and NIHR and consultancy and speaker fees from AstraZeneca, Roche, Edwards, and Bayer. MCP is Director of Global Clinical Trials Partners and has received research funding from BHF, NIHR, Boehringer Ingelheim, Roche, SQ Innovations, AstraZeneca, Novartis, Novo Nordisk, Medtronic, Boston Scientific, and Pharmacosmos; consulting fees from clinical trial committees or consulting for Abbott, Akero, Applied Therapeutics, Amgen, AnaCardio, Biosensors, Boehringer Ingelheim, Corteria, Novartis, AstraZeneca, Novo Nordisk, AbbVie, Bayer, Horizon Therapeutics, Foundry, Takeda, Cardiorentis, Pharmacosmos, Siemens, Eli Lilly, Vifor, New Amsterdam, Moderna, Teikoku, LIB Therapeutics, 3R Lifesciences, Reprieve, FIRE 1, Corvia, and Regeneron; and payment or honoraria from Boehringer Ingelheim, Novartis, AstraZeneca, Novo Nordisk, Pharmacosmos, Eli Lilly, and Vifor. JGFC has received grants from CSL-Vifor and Pharmacosmos; consulting fees from Biopeutics, Bayer, and Pharmacosmo; payment or honoraria from Pharmacosmos; and stock or stock options from Heartfelt and Viscardia. All other authors declare no competing interests.
