# Supplementary material for: Health inequalities and trends in heart failure diagnosis in primary care in England, 2000–21: a national retrospective cohort data-linkage study
Source: Lancet Prim Care. 2025 Dec;1(6):None. doi: 10.1016/j.lanprc.2025.100060 (PMC12728148; doi:10.1016/j.lanprc.2025.100060)
Supplement: Supplementary appendix [file mmc1.pdf]

THE LANCET
Primary Care

## Supplementary appendix

This appendix formed part of the original submission and has been peer reviewed.
We post it as supplied by the authors.

Supplement to: Lawson CA, Ali MR, McCann GP, et al. Health inequalities and trends in heart failure diagnosis in primary care in England, 2000–21: a national retrospective cohort data-linkage study. *Lancet Prim Care* 2025. https://doi.org/10.1016/j.lanprc.2025.100060

# Health inequalities and trends in heart failure diagnosis in primary care in England, 2000–21: a national retrospective cohort data-linkage study.

*Claire A Lawson, Mohammad R Ali, Gerry P McCann, Iain Squire, Francesco Zaccardi, Muhammad Rashid, Kelly Barber, Riaz Alaei Kalajahi, Christopher A Miller, Rhys Williams, Andrew L Clark, Mark C Petrie, Clare J Taylor, Jocelyn M Friday, Nathalie Conrad, John G F Cleland, Kamlesh Khunti*

A1. Supplementary methods: Joinpoint regression ..................................................................... 3

A2. Supplementary methods: Assessment of the robustness of case identification based on primary care coding ..................................................................................................... 4

A3 Figure: Flow chart of the patient selection process ........................................................... 7

A4 Table: Temporal trends in HF diagnostic care pathways in primary care during Covid-19, in those presenting with a recorded indication of heart failure (HF symptom or loop use) in primary care ........................................................................................................................ 8

A5 Figure: Trends in diagnosis during unplanned admission .................................................... 9

A6 Figure: Pathways to Outpatient diagnosis in patients with prior record of heart failure symptoms or loop diuretic use (restricted to 2015-2019) ....................................................... 10

A7 Figure: Pathways to diagnosis during unplanned hospitalisation for patients with prior record of heart failure symptoms or loop diuretic use (restricted to 2015-2019) ..................... 11

A8 Figure: Pathways to diagnosis in patients with no prior record of heart failure symptoms or loop diuretic use ........................................................................................................... 12

A9 Table: GRMT prescribing pre and post heart failure diagnosis ......................................... 14

A10 Table: Presentation with a possible indication of HF (symptom and/or loop diuretic use) in primary care during 5 years prior to HF diagnosis by sex, socioeconomic status, ethnicity and MLTCs ........................................................................................................................ 17

A11 Table: HF diagnostic care pathways in primary care, in patients with a recorded indication of heart failure, by sex, socioeconomic status, ethnicity and MLTCs (2015-2019; n=80,824) 18

A12 Table: Unadjusted group differences in tests, specialist referral, place of diagnosis and lag times, in patients with a recorded indication of heart failure (restricted to 2015-2019; n=80,824) ........................................................................................................................ 20

A13 Table: Adjusted group differences in tests, specialist referral, place of diagnosis and lag times, in patients with a recorded indication of heart failure (restricted to 2015-2019; n=80,824) ........................................................................................................................ 21

A14 Figure: Group differences in diagnostic tests, specialist review and diagnosis location .. 22

A15 Figure: Age adjusted survival in men and women following heart failure diagnosis, by diagnosis group ........................................................................................................ 23
A16 Table: Associations between diagnostic pathways and mortality for men and women with heart failure according to location of diagnosis and whether symptoms only or loop diuretic initiation preceded the diagnosis. Data are for 2015-2019 (men=63,127, women=56,228)..... 24
A17 Table: 1 year mortality comparison among all patients versus those remaining after excluding individuals with only a primary care code and no diagnostic test or GRMT prescription.................................................................................................................. 25
A18 Table: Adjusted group differences in tests, specialist referral and place of diagnosis, taking account of variation between general practices (restricted to 2015-2019; n=80,824) ... 26
A19 Table: Baseline characteristics comparing all patients with those that did not survive their index date ................................................................................................................ 27
A20 Table: Mortality comparing all patients with those that survived their index date........... 28

## A1. Supplementary methods: Joinpoint regression

This command applies piecewise linear regression, often referred to as “hockey stick” regression, within the framework of a logistic regression model. The approach assumes that the relationship between the log-odds of the dependent variable *(*e.g, proportion of NP tests*)* and the independent variable (i.e., year of diagnosis) is best represented by two distinct straight-line segments rather than a single continuous slope. The model estimates the slopes of these two segments (i.e., odds ratios for each increase in diagnosis year) and determines the breakpoint (also called the “knot”) where the slope changes direction or magnitude. This allows for capturing potential threshold effects or changes in the strength of association between year of diagnosis and the dependant variable.

See:

Gillis D, Edwards BPM. The utility of joinpoint regression for estimating population parameters given changes in population structure. Heliyon. 2019 Nov 19;5(11):e02515. doi:10.1016/j.heliyon.2019.e02515.

Rea F, Pagan E, Compagnoni M, Cantarutti A, Pugni P, Bagnardi V, et al. Joinpoint regression analysis with time-on-study as time-scale. Application to three Italian population-based cohort studies. Epidemiology Biostatistics and Public Health. 2017 July 4,;14(3).

**A2. Supplementary methods: Assessment of the robustness of case identification based on primary care coding**

In our study, 230,886 (56%) of patients received their first diagnostic code in an outpatient setting; 132,297 (57%) of these subsequently had a ICD-10 code for heart failure applied in their hospital record, leaving 98,589 (24%) with a primary care record only. Of these, 7,347 (7.5%) had a NP test, 27,573 (28%) had an echocardiogram and 31,157 (32%) were referred for a cardiology specialist review during the 6 months prior to their HF diagnosis in primary care. This left 49,285 (12%) patients with a primary care code only (without an ICD 10 code or diagnostic test recorded in primary care). 31,681 (64%) of these patients were prescribed guideline-recommended medical therapy (GRMT) for HF (beta blocker or a renin–angiotensin–aldosterone system inhibitor) within the 6 months prior to or after diagnosis. The remaining number patients with a primary care code only (without an ICD 10 code, diagnostic test or GRMT) was 17,604 (4.3%).

To assess the robustness of case identification based on primary care coding, we performed two sensitivity analyses:

First, we compared our findings with a published study by Koudstaal et al. (using CPRD data from 1997-2010, linked to hospital, death registry, and MINAP data), which compared outcomes among patients with (1) a primary care heart failure code only, (2) both primary and secondary care codes, and (3) a secondary care code only. They found that mortality in the HF primary care only group was substantially higher than in the general population and comparable to the HF primary and secondary care group, supporting the clinical relevance of primary care coding for HF. We repeated this approach and found that, while overall survival has improved since Koudstaal's study in 2010 (especially in older patients and those with secondary care records) survival in the primary care only group remained poor, markedly lower than in the general population (see table below), and only slightly better than in the combined primary and secondary code group (see figure below).

These findings suggest that even when coded only in primary care, heart failure is associated with substantially elevated mortality, reinforcing the validity of using primary care codes for case identification in this context.

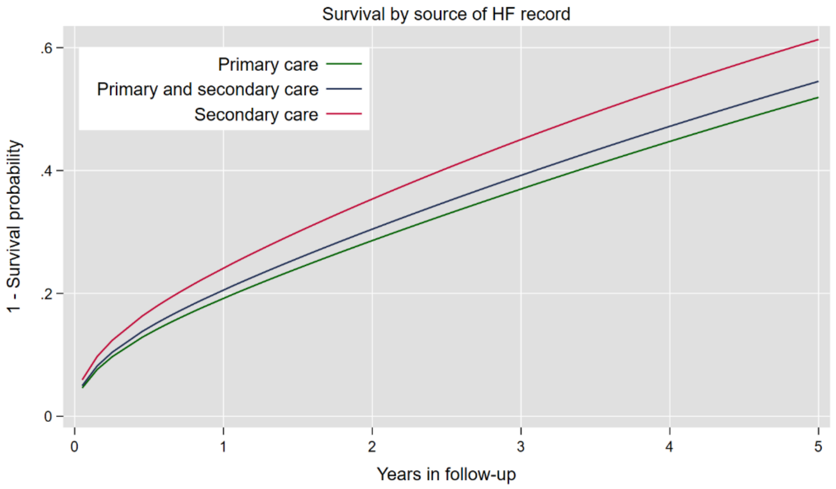

Sensitivity analysis: Five-year survival estimates between patients with heart failure recorded in primary care, hospital admissions, or both compared with a prior study by Koudstaal et al.

| | | Source of HF record (HF population) | | | | | |
|---|---|---|---|---|---|---|---|
| | | CPRD | | CPRD and HES | | HES | |
| | Age- and sex-matched general population | Primary care record of HF but never hospitalized for HF | | Primary care record of HF and hospitalized at least once for HF | | Hospitalized for HF without primary care record of HF | |
| | Koustall et al | Koustall et al | Current analysis | Koustall et al | Current analysis | Koustall et al | Current analysis |
| No. of patients | 77 857 | 23 547 | 28,088 | 23 681 | 146,208 | 30 629 | 237,877 |
| Age < 55 years | 99.7% (99.5, 99.8) | 81.7% (79.1, 84.3) | 81.7% (81.2,82.2) | 69.7% (65.8, 73.9) | 75.7% (74.7,76.7) | 56.9% (53.7, 60.3) | 71.2% (69.9,72.4) |
| Age 55–75 years | 96.5% (96.3, 96.7) | 62.1% (60.9, 63.4) | 66.8% (66.5,67.0) | 56.0% (54.7, 57.3) | 62.2% (61.9,62.6) | 40.0% (38.6, 41.3) | 56.8% (56.2,57.4) |
| Age > 75 years | 75.4% (74.9, 75.8) | 32.7% (31.8, 33.6) | 35.3% (35.1,35.5) | 32.4% (31.6, 33.2) | 33.9% (33.6,34.2) | 13.2% (12.7, 13.8) | 26.9% (26.5,27.3) |
| Men | 88.7% (88.4, 89.0) | 44.2% (43.2, 45.3) | 55.2% (54.8,55.6) | 41.3% (40.3, 42.3) | 51.8% (51.5,52.0) | 24.5% (23.6, 25.4) | 47.5% (47.1,47.9) |
| Women | 87.5% (87.2, 87.8) | 43.6% (42.6, 44.6) | 51.5% (51.1,52.0) | 38.4% (37.4, 39.3) | 47.25 (46.9,47.5) | 19.2% (18.5, 20.0) | 41.7% (41.2,42.0) |
| Total | 88.1% (87.9, 88.3) | 43.9% (43.2, 44.6) | 49.7% (49.5,49.8) | 39.8% (39.2, 40.5) | 45.8% (45.6,46.0) | 21.7% (21.1, 22.2) | 40.4% (39.9,40.6) |

CPRD, Clinical Practice Research Datalink; HES, Hospital Episodes Statistics; HF, heart failure.

Koudstaal S, Pujades-Rodriguez M, Denaxas S, Gho JMIH, Shah AD, Yu N, Patel RS, Gale CP, Hoes AW, Cleland JG, Asselbergs FW, Hemingway H. Prognostic burden of heart failure recorded in primary care, acute hospital admissions, or both: a population-based linked electronic health record cohort study in 2.1 million people. Eur J Heart Fail. 2017 Sep;19(9):1119-1127. doi: 10.1002/ejhf.709. Epub 2016 Dec 23. PMID: 28008698; PMCID: PMC5420446.

Second, we refined the primary care group by further stratifying patients into two categories based on evidence of diagnostic investigations or guideline-directed medical therapy (GDMT): (ia) primary care code only with no diagnostic test or GDMT prescription, (ib) primary care code only with at least one diagnostic test or GDMT prescription and compared age adjusted survival with the remining two groups with (ii) primary care and secondary care codes and (iii) secondary care codes only.

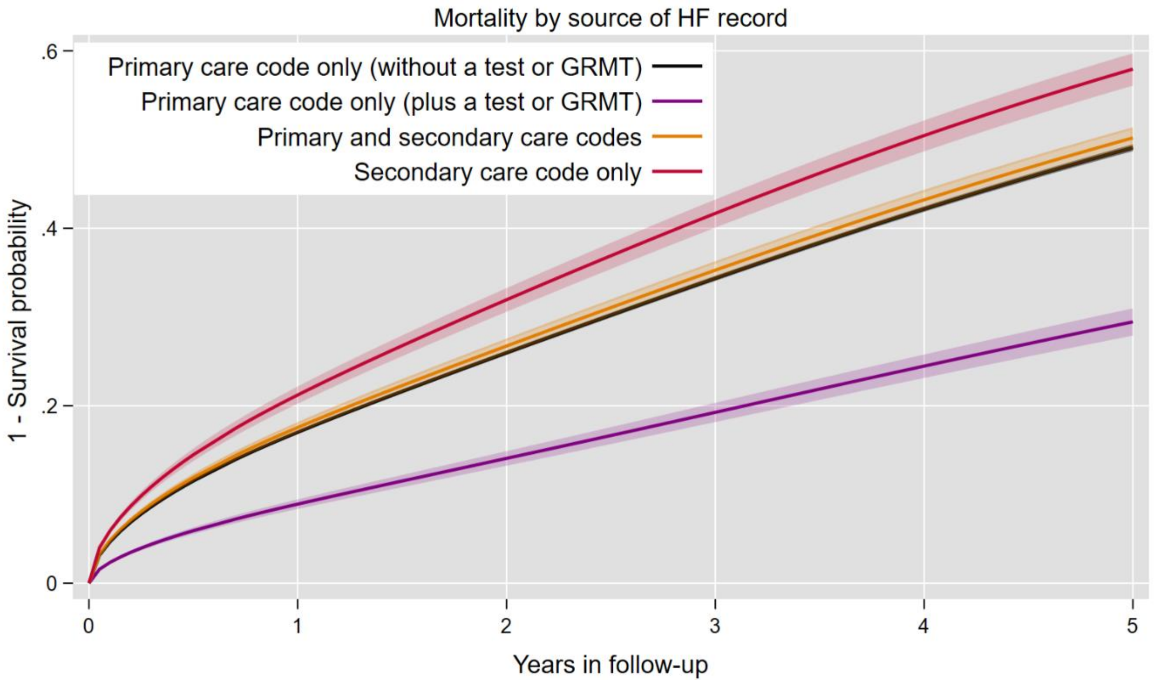


As expected, the highest mortality rates were observed in the group diagnosed during an unplanned hospitalisation (secondary care code only). Patients with a primary care code only, without a recorded diagnostic test or GRMT prescription (n=17,604; 4.3%), had mortality rates similar to those with both primary and secondary care codes for HF. The lowest mortality was seen in the primary care only group with evidence of at least one diagnostic test in the 6 months leading up to diagnosis or a GRMT prescription at the time immediately before or after diagnosis. (n=80,985; 20%).

### A3 Figure:  Flow chart of the patient selection process

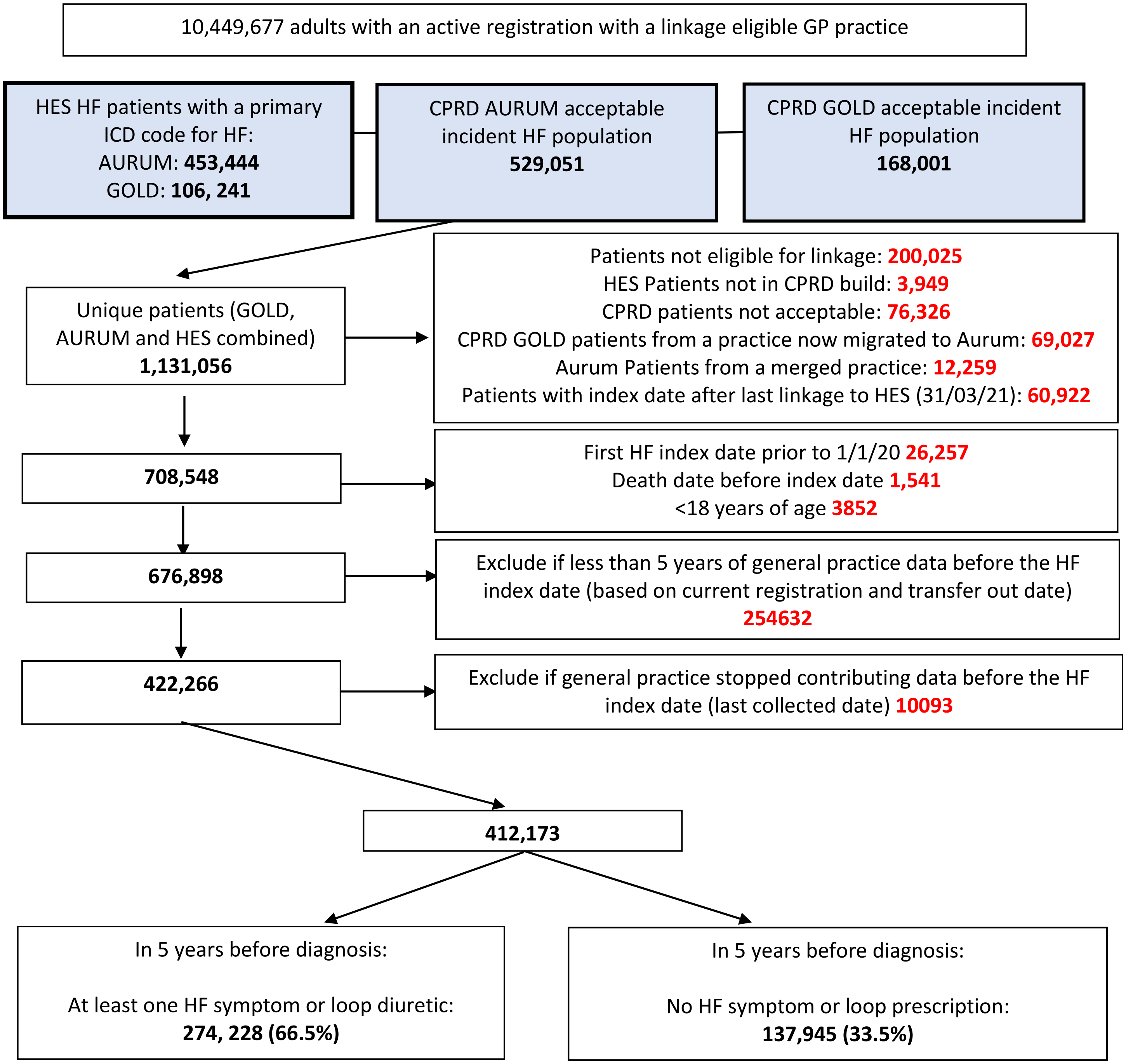

**A4 Table: Temporal trends in HF diagnostic care pathways in primary care during Covid-19, in those presenting with a recorded indication of heart failure (HF symptom or loop use) in primary care**

| **Impact of Covid** | 2019 (n=17,153) | 2020 (n=14,085) | Absolute difference in percentage between 2019 to 2020 |
|---|---|---|---|
| | Percentage with 95% CI | | |
| NP | 9.8 (9.4, 10.3) | 8.4 (8.0, 8.9) | -1.4 (-2.0, -0.7) |
| Echocardiogram | 19.8 (19.2, 20.4) | 17.7 (17.0, 18.3) | -2.1 (-3.0, -1.2) |
| Specialist referral | 36.1 (35.4, 36.9) | 33.0 (32.3, 33.8) | -3.1 (-4.2, -2.0) |
| Any pathway | 47.9 (47.1, 48.6) | 43.4 (42.6, 44.3) | -4.4 (-5.6, -3.3) |
| No pathway | 52.1 (51.4, 52.9) | 56.6 (55.7, 57.4) | 4.4 (5.6, 3.3) |
| Diagnosis during unplanned hospitalisation | 47.7 (46.9, 48.4) | 51.9 (51.1, 52.7) | 4.2 (3.1, 5.3) |

NP, natriuretic peptide. Any pathway: at least one of natriuretic peptide (NP) test, echocardiogram or specialist review, recorded in the primary care record in the 6 months prior to HF diagnosis. No pathway: None of NP, echocardiogram or specialist review, recorded in the primary care record in the 6 months prior to HF diagnosis.  All diagnostic processes are estimated at the mean population age (78 years) and reported as percentages with 95% CI.

**A5 Figure: Trends in diagnosis during unplanned admission**

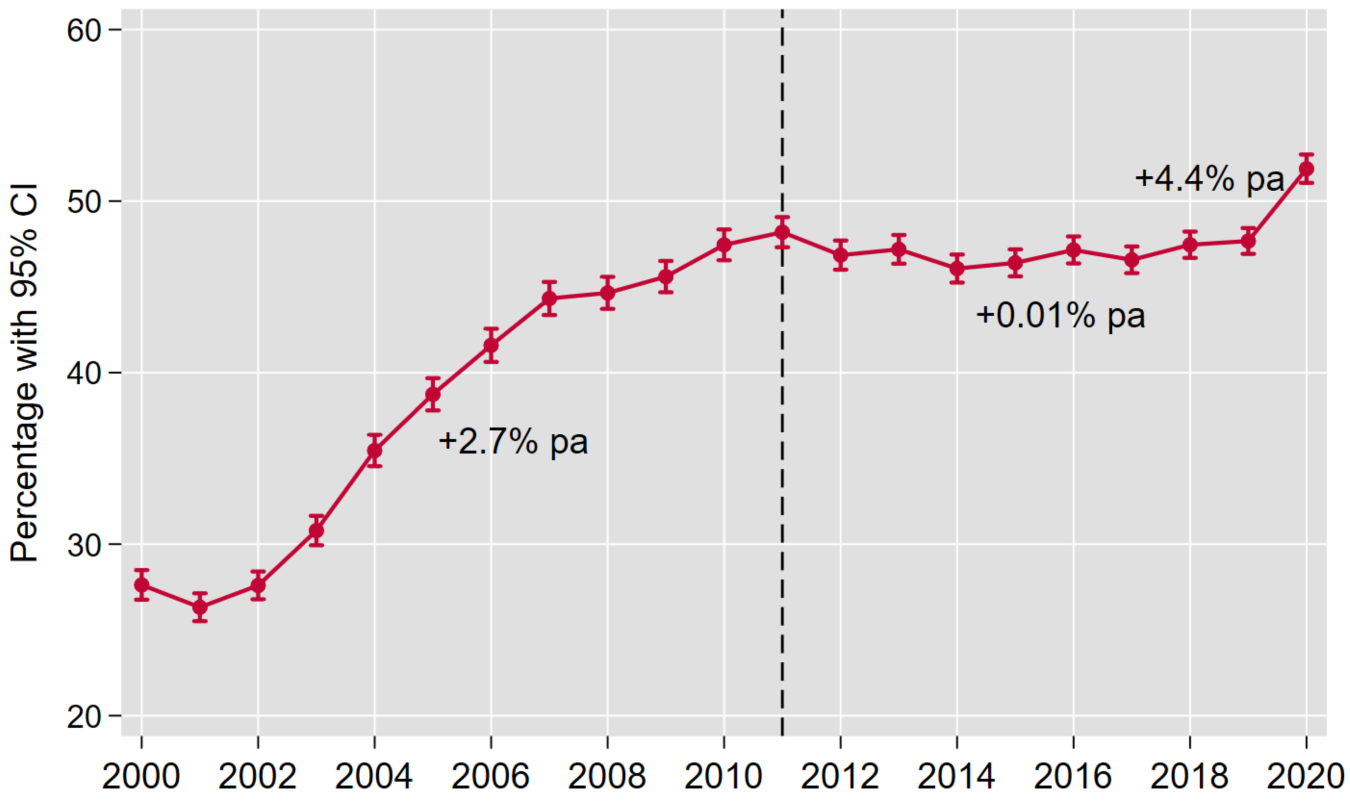


Trends in diagnosis during unplanned admission by calendar year of diagnosis. Pa, per annum. Models were restricted to patients with a prior indication of heart failure (symptom or loop diuretic use; n=274,228). Percentages with 95% CI were estimated using margins predicted at age 78, after fitting a logistic model including an interaction between year and age. Dotted line indicates a significant change in the trend line5

**A6 Figure: Pathways to <u>Outpatient</u> diagnosis in patients with prior record of heart failure symptoms or loop diuretic use (restricted to 2015-2019)**

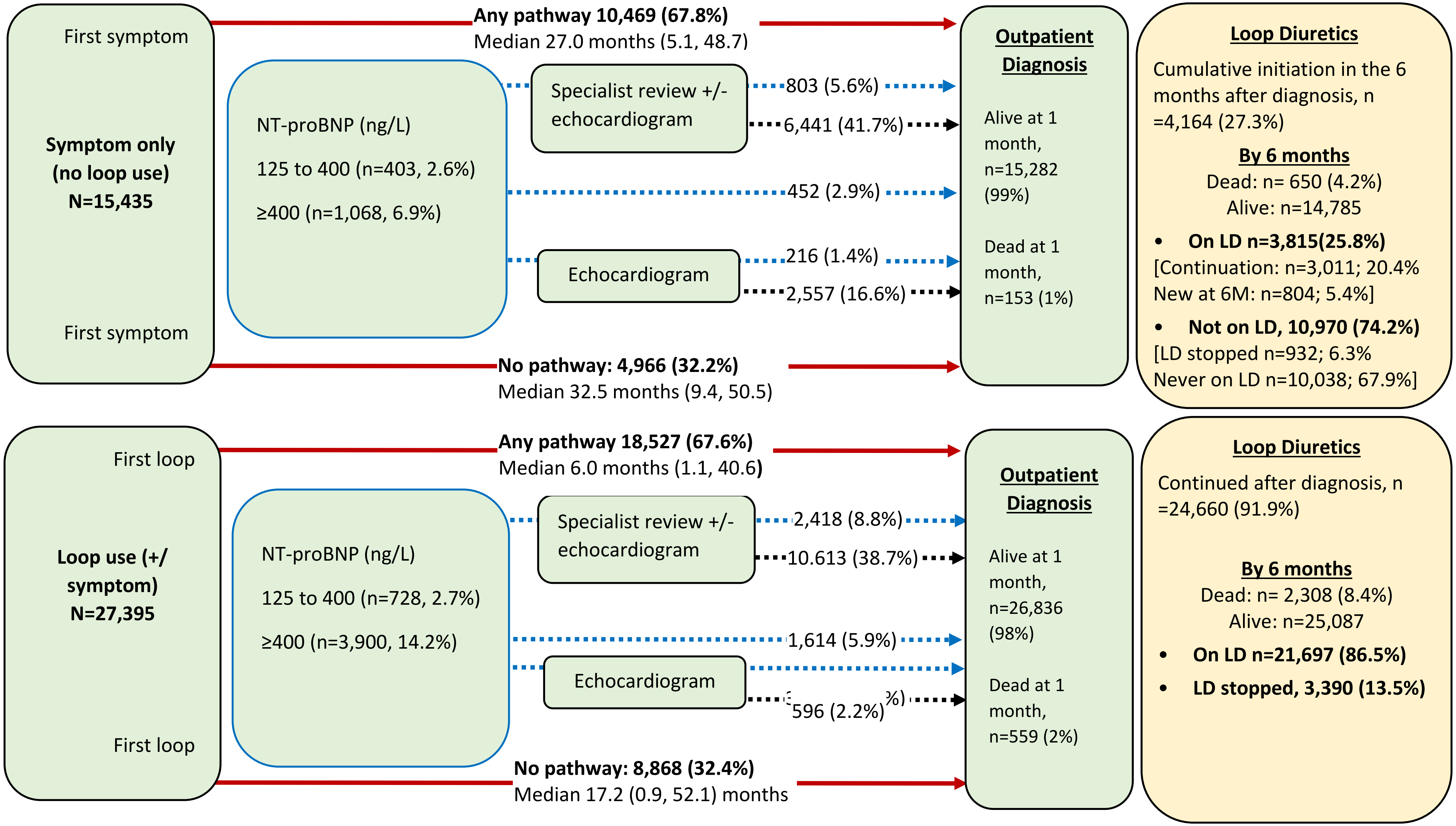

**A7 Figure: Pathways to <u>diagnosis during unplanned hospitalisation</u> for patients with prior record of heart failure symptoms or loop diuretic use (restricted to 2015-2019)**

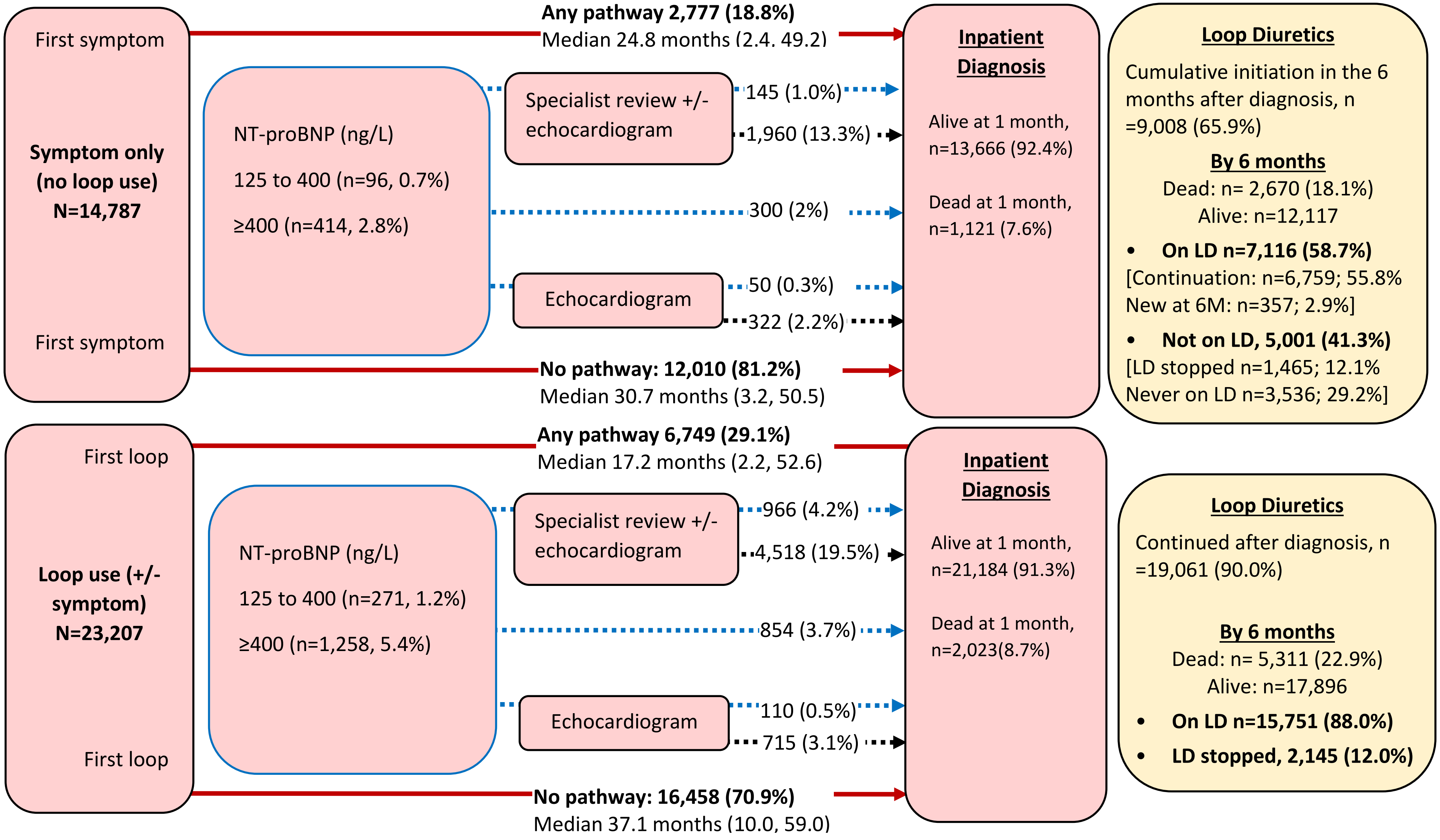

**A8 Figure: Pathways to diagnosis in patients with <u>no prior record</u> of heart failure symptoms or loop diuretic use**

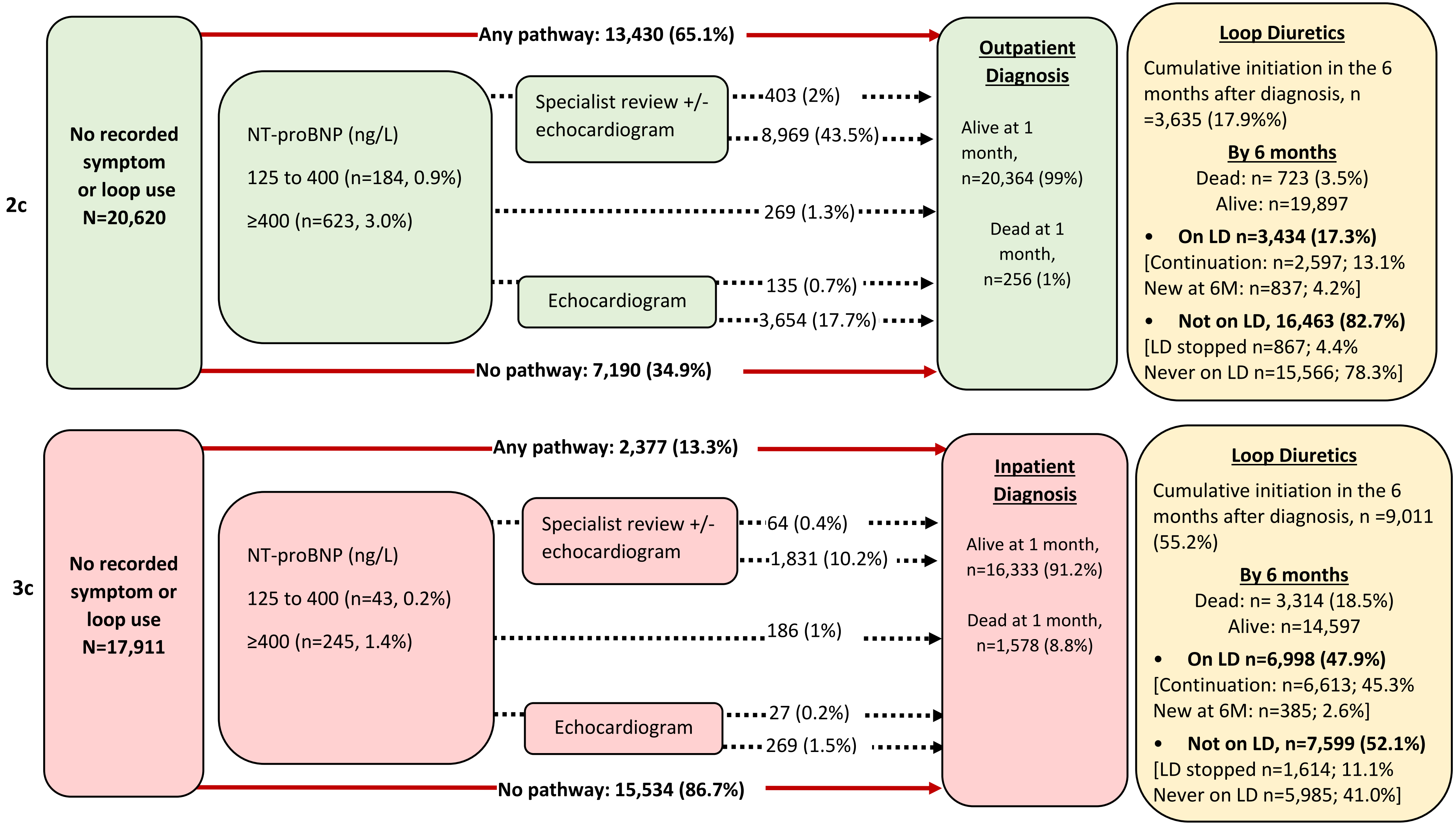

A6, 7 & 8 Figures: Pathways to diagnosis recorded in primary care in the 6 months prior to diagnosis, in patients diagnosed with HF during the recent years prior to Covid-19 (2015-2019; n=119,335). NT-proBNP, N-terminal pro BNP; ng/L, nanograms per litre. Any pathway: at least one of NT-proBNP test ≥400 or between 125 and 400, echocardiogram or specialist review. No pathway: None of NT-proBNP, echocardiogram or specialist review.

## A9 Table: GRMT prescribing pre and post heart failure diagnosis

| | OUTPATIENT DIAGNOSIS | | | | | |
|---|---|---|---|---|---|---|
| | **Any GRMT** | **BB** | **RAAS** | **MRA** | **ARNi** | **SGLT2i** |
| **Pathway 2a (i):** Symptom only WITH a prior diagnostic pathway in primary care: n=10,469 (pre diagnosis), 10,434 (1 month post discharge) | | | | | | |
| Already prescribed at 6-months prior to diagnosis | 5,598 (53) | 2,395 (23) | 4,636 (44) | 181 (2) | 3 (0) | 56 (1) |
| Newly prescribed 6-months prior to diagnosis | 1,235 (12) | 1,403 (13) | 859 (8) | 192 (2) | 3 (0) | 22 (0) |
| Continued after diagnosis | 6,510 (62) | 3,507 (34) | 5,080 (49) | 306 (3) | 6 (0) | 60 (1) |
| Newly prescribed after diagnosis | 2,085 (20) | 2,418 (23) | 2,361 (23) | 1,224 (12) | 80 (1) | 28 (0) |
| Discontinued after diagnosis | 155 (1) | 186 (2) | 239 (2) | 48 (0) | 0 (0) | 8 () |
| **Pathway 2a (ii):** Symptom only WITHOUT a prior diagnostic pathway in primary care: n=4,966 (pre diagnosis), 4,848 (1 month post discharge) | | | | | | |
| Already prescribed at 6-months prior to diagnosis | 2,604 (52) | 1,043 (21) | 2,216 (45) | 88 (2) | 0 (0) | 40 (1) |
| Newly prescribed 6-months prior to diagnosis | 278 (6) | 296 (6) | 169 (3) | 55 (1) | 0 (0) | 13 (0) |
| Continued after diagnosis | 2,627 (54) | 1,206 (25) | 2,101 (43) | 104 (2) | 0 (0) | 40 (1) |
| Newly prescribed after diagnosis | 892 (18) | 1,009 (21) | 891 (18) | 456 (9) | 25 (1) | 8 (0) |
| Discontinued after diagnosis | 113 (2) | 72 (1) | 151 (3) | 20 (0) | 0 (0) | 4 (0) |
| **Pathway 2b(i):** Loop WITH a prior diagnostic pathway in primary care: n=18,527 (pre diagnosis), 18,285 (1 month post discharge) | | | | | | |
| Already prescribed at 6-months prior to diagnosis | 12,497 (67) | 5,858 (32) | 10,522 (57) | 730 (4) | 7 (0) | 95 (1) |
| Newly prescribed 6-months prior to diagnosis | 2,338 (13) | 3,085 (17) | 1,705 (9) | 989 (5) | 15 (0) | 28 (0) |
| Continued after diagnosis | 14,011 (77) | 8,288 (45) | 10,901 (60) | 1,389 (8) | 18 (0) | 82 (0) |
| Newly prescribed after diagnosis | 2,291 (13) | 3,195 (17) | 2,893 (16) | 3,039 (17) | 151 (1) | 39 (0) |
| Discontinued after diagnosis | 382 (2) | 406 (2) | 771 (4) | 202 (1) | 3 (0) | 24 (o) |
| **Pathway 2b(ii):** Loop WITHOUT a prior diagnostic pathway in primary care: n=8,868 (pre diagnosis), 8,551 (1 month post discharge) | | | | | | |
| Already prescribed at 6-months prior to diagnosis | 6,028 (68) | 2,724 (31) | 5,034 (57) | 394 (4) | 2 (0) | 41 (0) |
| Newly prescribed 6-months prior to diagnosis | 586 (7) | 716 (8) | 329 (4) | 222 (3) | 1 (0) | 13 (0) |
| Continued after diagnosis | 5,947 (70) | 3,107 (36) | 4,552 (53) | 445 (5) | 2 (0) | 31 (0) |
| Newly prescribed after diagnosis | 1,082 (13) | 1,374 (16) | 1,124 (13) | 1,072 (13) | 34 (0) | 11 (0) |
| Discontinued after diagnosis | 286 (3) | 131 (2) | 452 (5) | 97 (1) | 1 (0) | 14 0() |
| **Pathway 2c(i):** No symptom or Loop WITH a prior diagnostic pathway in primary care: n=13,430 (pre diagnosis), 13,383 (1 month post discharge) | | | | | | |
| Already prescribed at 6-months prior to diagnosis | 6,367 (47) | 2,688 (20) | 5,426 (40) | 209 (2) | 1 (0) | 99 (1) |
| Newly prescribed 6-months prior to diagnosis | 2,206 (16) | 2,416 (18) | 1,457 (11) | 318 (2) | 7 (0) | 26 (0) |
| Continued after diagnosis | 8,138 (61) | 4,741 (35) | 6,346 (47) | 458 (3) | 6 (0) | 90 (1) |
| Newly prescribed after diagnosis | 2,989 (22) | 3,462 (26) | 3,439 (26) | 1,440 (11) | 95 (1) | 41 (0) |
| Discontinued after diagnosis | 230 (2) | 247 (2) | 307 (2) | 40 (0) | 2 (0) | 24 (0) |
| **Pathway 2c(ii):** No symptom or Loop WITHOUT a prior diagnostic pathway in primary care: n=7,190 (pre diagnosis), 6,981 (1 month post discharge) | | | | | | |
| Already prescribed at 6-months prior to diagnosis | 3,465 (48) | 1,395 (19) | 2,972 (41) | 109 (2) | 0 (0) | 51 (1) |
| Newly prescribed 6-months prior to diagnosis | 500 (7) | 521 (7) | 309 (4) | 77 (1) | 0 (0) | 18 (0) |
| Continued after diagnosis | 3,610 (52) | 1,744 (25) | 2,875 (41) | 162 (2) | 0 (0) | 58 (1) |

| | | | | | | |
|---|---|---|---|---|---|---|
| Newly prescribed after diagnosis | 1,538 (22) | 1,818 (26) | 1,516 (22) | 738 (11) | 32 (0) | 23 (0) |
| Discontinued after diagnosis | 158 (2) | 83 (1) | 222 (3) | 13 (0) | 0 (0) | 7 (0) |
| **INPATIENT DIAGNOSIS** | | | | | | |
| **Pathway 3a(i):** Symptom only WITH a prior diagnostic pathway in primary care: n=2,777 (pre diagnosis), 2,648 (1 month post discharge) | | | | | | |
| Already prescribed at 6-months prior to diagnosis | 1,631 (59) | 722 (26) | 1,353 (49) | 59 (2) | 1 (0) | 18 (1) |
| Newly prescribed 6-months prior to diagnosis | 316 (11) | 445 (16) | 178 (6) | 48 (2) | 0 (0) | 5 (0) |
| Continued after diagnosis | 1,667 (63) | 965 (36) | 1,148 (43) | 67 (3) | 1 (0) | 10 (0) |
| Newly prescribed after diagnosis | 571 (22) | 830 (31) | 590 (22) | 638 (24) | 25 (1) | 9 (0) |
| Discontinued after diagnosis | 122 (5) | 119 (4) | 232 (9) | 27 (1) | 0 (0) | 9 (0) |
| **Pathway 3a(ii):** Symptom only WITHOUT a prior diagnostic pathway in primary care: n=12,010 (pre diagnosis), 11,018 (1 month post discharge) | | | | | | |
| Already prescribed at 6-months prior to diagnosis | 6,065 (50) | 2,099 (17) | 5,166 (43) | 169 (1) | 0 (0) | 82 (1) |
| Newly prescribed 6-months prior to diagnosis | 430 (4) | 493 (4) | 222 (2) | 52 (0) | 1 (0) | 20 (0) |
| Continued after diagnosis | 5,208 (47) | 2,121 (19) | 3,852 (35) | 114 (1) | 0 (0) | 52 (0) |
| Newly prescribed after diagnosis | 3,363 (31) | 4,419 (40) | 2,887 (26) | 2,470 (22) | 86 (1) | 24 (0) |
| Discontinued after diagnosis | 515 (5) | 192 (2) | 824 (7) | 56 (1) | 1 (0) | 33 (0) |
| **Pathway 3b(i)::** Loop WITH a prior diagnostic pathway in primary care: n=6,749 (pre diagnosis), 6,297 (1 month post discharge) | | | | | | |
| Already prescribed at 6-months prior to diagnosis | 4,709 (70) | 2,238 (33) | 3,920 (58) | 361 (5) | 3 (0) | 51 (1) |
| Newly prescribed 6-months prior to diagnosis | 760 (11) | 1,090 (16) | 464 (7) | 413 (6) | 3 (0) | 3 (0) |
| Continued after diagnosis | 4,550 (72) | 2,691 (43) | 3,083 (49) | 453 (7) | 4 (0) | 30 (0) |
| Newly prescribed after diagnosis | 714 (11) | 1,225 (19) | 716 (11) | 1,389 (22) | 38 (1) | 16 (0) |
| Discontinued after diagnosis | 458 (7) | 342 (5) | 832 (13) | 216 (3) | 1 (0) | 17 (0) |
| **Pathway 3b(ii):** Loop WITHOUT a prior diagnostic pathway in primary care: n=16,458 (pre diagnosis), 14,887 (1 month post discharge) | | | | | | |
| Already prescribed at 6-months prior to diagnosis | 11,007 (67) | 4,770 (29) | 8,959 (54) | 866 (5) | 2 (0) | 103 (1) |
| Newly prescribed 6-months prior to diagnosis | 706 (4) | 887 (5) | 360 (2) | 337 (2) | 0 (0) | 14 (0) |
| Continued after diagnosis | 9,227 (62) | 4,542 (31) | 6,359 (43) | 679 (5) | 1 (0) | 64 (0) |
| Newly prescribed after diagnosis | 1,863 (13) | 2,927 (20) | 1,466 (10) | 2,331 (16) | 40 (0) | 27 (0) |
| Discontinued after diagnosis | 1,100 (7) | 491 (3) | 1,683 (11) | 298 (2) | 0 (0) | 29 (0) |
| **Pathway 3c(i):** No symptom or Loop WITH a prior diagnostic pathway in primary care: n=2,377 (pre diagnosis), 2,251 (1 month post discharge) | | | | | | |
| Already prescribed at 6-months prior to diagnosis | 1,342 (56) | 586 (25) | 1,119 (47) | 50 (2) | 1 (0) | 20 (1) |
| Newly prescribed 6-months prior to diagnosis | 323 (14) | 441 (19) | 148 (6) | 39 (2) | 0 (0) | 7 (0) |
| Continued after diagnosis | 1,419 (63) | 865 (38) | 958 (43) | 64 (3) | 1 (0) | 17 (1) |
| Newly prescribed after diagnosis | 488 (22) | 745 (33) | 538 (24) | 549 (24) | 17 (1) | 6 (0) |
| Discontinued after diagnosis | 108 (5) | 92 (4) | 176 (8) | 16 (1) | 0 (0) | 5 (0) |
| **Pathway 3c(ii):** No symptom or Loop WITHOUT a prior diagnostic pathway in primary care: n=15,534 (pre diagnosis), 14,082 (1 month post discharge) | | | | | | |
| Already prescribed at 6-months prior to diagnosis | 6,806 (44) | 2,259 (15) | 5,884 (38) | 160 (1) | 2 (0) | 91 (1) |
| Newly prescribed 6-months prior to diagnosis | 485 (3) | 511 (3) | 245 (2) | 42 (0) | 0 (0) | 15 (0) |
| Continued after diagnosis | 5,669 (40) | 2,174 (15) | 4,238 (30) | 108 (1) | 2 (0) | 71 (1) |
| Newly prescribed after diagnosis | 5,113 (36) | 6,562 (47) | 4,418 (31) | 3,695 (26) | 121 (1) | 44 (0) |

| | | | | | | |
|---|---|---|---|---|---|---|
| Discontinued after diagnosis | 659 (5) | 261 (2) | 1,025 (7) | 44 (0) | 0 (0) | 22 (0) |

*Restricted to latest data (2015-2019; n=119,355). GRMT; guideline recommended medical therapy (any). BB; beta blocker, RAAS; renin–angiotensin–aldosterone system inhibitors (comprising angiotensin converting enzyme inhibitors and angiotensin II receptor blockers); ARNi; angiotensin receptor/neprilysin inhibitor, SGLT2i; sodium/glucose cotransporter 2 inhibitors, MRA; mineralocorticoid receptor antagonists. Prescribed drugs were identified by at least one prescription in the relevant time window.

**A10 Table: Presentation with a possible indication of HF (symptom and/or loop diuretic use) in primary care during 5 years prior to HF diagnosis by sex, socioeconomic status, ethnicity and MLTCs**

| | Male N=217,998 | Female N=194,175 | Most affluent N=79,390 | Most deprived N=82,611 | <4 MLTCs N=197,885 | ≥4 MLTCs N=214,288 |
|---|---|---|---|---|---|---|
| **Consultation history in 5 years prior** | | | | | | |
| Symptom consultation | 99,284 (45.5) | 97,230 (50.1) | 37,589 (47.3) | 39,785 (48.2) | 77,901 (39.4%) | 118,613 (55.4%) |
| • Shortness of breath | 83,954 (38.5) | 77,284 (39.8) | 30,365 (38.2) | 33,292 (40.3) | 61,738 (31.2%) | 99,500 (46.4%) |
| • Fatigue | 17,779 (8.2) | 23,115 (11.9) | 8,189 (10.3) | 7,854 (9.5) | 15,504 (7.8%) | 25,390 (11.8%) |
| • Ankle swelling | 17,274 (7.9) | 23,284 (12.0) | 7,760 (9.8) | 8,265 (10.0) | 15,019 (7.6%) | 25,539 (11.9%) |
| Symptom only (no loop diuretic use) | 45,237 (20.8) | 41,308 (21.3) | 16,479 (20.8) | 17,994 (21.8) | 39,614 (20.0%) | 46,931 (21.9%) |
| Prescribed a loop diuretic in 5 years prior | 92,608 (42.5) | 95,075 (49.0) | 35,892 (45.2) | 37,263 (45.1) | 75,430 (38.1%) | 112,253 (52.4%) |
| Symptom or loop diuretic use | 137,845 (63.2) | 136,383 (70.2) | 52,371 (66.0) | 55,257 (66.9) | 115,044 (58.1%) | 159,184 (74.3%) |
| | **White N=375,808** | **South Asian N=11,644** | **Black N=6,994** | | | |
| **Consultation history in 5 years prior** | | | | | | |
| Symptom consultation | 181,926 (48.4) | 5,364 (46.1) | 3,052 (43.6) | | | |
| • Shortness of breath | 150,257 (40.0) | 3,912 (33.6) | 2,193 (31.4) | | | |
| • Fatigue | 37,214 (9.9) | 1,701 (14.6) | 762 (10.9) | | | |
| • Ankle swelling | 37,271 (9.9) | 1,265 (10.9) | 785 (11.2) | | | |
| Symptom only (no loop diuretic use) | 79,756 (31.7) | 2,595 (35.7) | 1,572 (37.4) | | | |
| Prescribed a loop diuretic in 5 years prior | 172,101 (45.8) | 4,669 (40.1) | 2,634 (37.7) | | | |
| Symptom or loop diuretic use | 251,857 (67.0) | 7,264 (62.4) | 4,206 (60.1) | | | |

Data are presented as N (%) or median (1st, 3rd quartiles).

## A11 Table: HF diagnostic care pathways in primary care, in patients with a recorded indication of heart failure, by sex, socioeconomic status, ethnicity and MLTCs (2015-2019; n=80,824)

| | **Male** | **Female** | **Most affluent** | **Most deprived** | **<4 MLTCs** | **≥4 MLTCs** |
|---|---|---|---|---|---|---|
| | **N=40,235** | **N=40,589** | **N=16,011** | **N=16,132** | **N=27,273** | **N=53,551** |
| **Diagnostic testing and cardiology review in 5 years prior to HF diagnosis** | | | | | | |
| At least one NP | 5,078 (12.6) | 5,001 (12.3) | 2,221 (13.9) | 1,783 (11.1) | 4,051 (14.9%) | 6,028 (11.3%) |
| Highest NT-proBNP value in ng/L ng/L | | | | | | |
| • <125 | 306 (6.9) | 328 (7.5) | 120 (6.4) | 148 (9.3) | 323 (9.1%) | 311 (6.0%) |
| • 125 to 400 | 692 (15.7) | 806 (18.5) | 327 (17.4) | 283 (17.9) | 649 (18.3%) | 849 (16.3%) |
| • ≥400 | 3,418 (77.4) | 3,222 (74.0) | 1,434 (76.2) | 1,152 (72.8) | 2,584 (72.7%) | 4,056 (77.8%) |
| At least one echocardiogram | 8,366 (20.8) | 7,620 (18.8) | 3,417 (21.3) | 2,753 (17.1) | 6,572 (24.1%) | 9,414 (17.6%) |
| At least one cardiology specialist review | 15,108 (37.5) | 12,696 (31.3) | 5,868 (36.6) | 5,306 (32.9) | 9,887 (36.3%) | 17,917 (33.5%) |
| **Diagnostic pathways prior to HF diagnosis** | | | | | | |
| Any pathway | 20,377 (50.6) | 18,145 (44.7) | 8,066 (50.4) | 7,216 (44.7) | 14,268 (52.3%) | 24,254 (45.3%) |
| No pathway | 19,858 (49.4) | 22,444 (55.3) | 7,945 (49.6) | 8,916 (55.3) | 13,005 (47.7%) | 29,297 (54.7%) |
| **Lag times in patients from a first indication of HF; Median (1st, 3rd quartile)** | | | | | | |
| Time from first HF symptom to diagnosis | 29.2 (4.7-50.4) | 32.6 (7.6-50.5) | 27.5 (4.3-48.7) | 35.9 (9.3-51.9) | 18.1 (2.0-44.1) | 36.9 (11.0-52.1) |
| Time from first loop diuretic to diagnosis | 13.3 (1.4-48.6) | 25.9 (2.6-57.6) | 16.2 (1.5-50.9) | 23.7 (2.5-57.5) | 4.7 (0.8-36.5) | 27.0 (3.3-57.7) |
| **Diagnosis during unplanned hospitalisation** | 17,872 (44.4) | 20,122 (49.6) | 6,935 (43.3) | 8,210 (50.9) | 9,886 (36.2%) | 28,108 (52.5%) |

| | **White** | **South Asian** | **Black** |
|---|---|---|---|
| | **N=75,341** | **N=2,869** | **N=1,555** |
| **Diagnostic testing and cardiology review in 5 years prior to HF diagnosis** | | | |
| At least one BNP | 9,540 (12.7) | 268 (9.3) | 141 (9.1) |
| Highest BNP value in ng/L | | | |
| • <125 | 584 (7.0) | 23 (10.9) | 16 (16.0) |
| • 125 to 400 | 1,415 (17.0) | 51 (24.2) | 11 (11.0) |
| • ≥400 | 6,346 (76.0) | 137 (64.9) | 73 (73.0) |
| At least one echocardiogram | 15,003 (19.9) | 477 (16.6) | 287 (18.5) |
| At least one cardiology specialist review | 25,700 (34.1) | 1,157 (40.3) | 580 (37.3) |

| | | | |
|---|---|---|---|
| **Diagnostic pathways prior to HF diagnosis** | | | |
| Any pathway | 35,791 (47.5) | 1,444 (50.3) | 757 (48.7) |
| No pathway | 39,550 (52.5) | 1,425 (49.7) | 798 (51.3) |
| **Lag times in patients from a first indication of HF; Median (1st, 3rd quartile)** | | | |
| Time from first HF symptom to diagnosis | 31.5 (5.9-50.7) | 27.6 (7.4-47.3) | 24.4 (5.9-45.0) |
| Time from first loop diuretic to diagnosis | 19.6 (1.8-54.5) | 21.6 (2.9-51.7) | 16.4 (1.8-52.7) |
| **Diagnosis during unplanned hospitalisation** | 35,467 (47.1) | 1,365 (47.6) | 699 (45.0) |

NP, natriuretic peptide; NT-proBNP, N-terminal pro BNP; ng/L, nanograms per litre; HF, heart failure. Any pathway: at least one of natriuretic peptide (NP) test, echocardiogram or specialist review. No pathway: None of NP, echocardiogram or specialist review. Data are presented as median (1st, 3rd quartiles) for continuous measures, and n (%) for categorical measures.

**A12 Table: Unadjusted group differences in tests, specialist referral, place of diagnosis and lag times, in patients with a recorded indication of heart failure (restricted to 2015-2019; n=80,824)**

| | NP | Echocardiogram | Specialist referral | Any pathway | No pathway | Diagnosis during unplanned hospitalisation |
|---|---|---|---|---|---|---|
| | **OR (95% CI)** | | | | | |
| Male | Ref | Ref | Ref | Ref | Ref | Ref |
| Female | 0.97 (0.93, 1.01) | 0.88 (0.85, 0.91) | 0.76 (0.74, 0.78) | 0.79 (0.77, 0.81) | 1.27 (1.23, 1.30) | 1.23 (1.20, 1.26) |
| Most affluent | Ref | Ref | Ref | Ref | Ref | Ref |
| Most deprived | 0.77 (0.72, 0.82) | 0.76 (0.72, 0.80) | 0.85 (0.81, 0.89) | 0.80 (0.76, 0.83) | 1.25 (1.20, 1.31) | 1.36 (1.30, 1.42) |
| White | Ref | Ref | Ref | Ref | Ref | Ref |
| South Asian | 0.71 (0.63, 0.81) | 0.80 (0.73, 0.89) | 1.31 (1.21, 1.41) | 1.12 (1.04, 1.21) | 0.89 (0.83, 0.96) | 1.02 (0.95, 1.10) |
| Black | 0.69 (0.58, 0.82) | 0.91 (0.80, 1.04) | 1.15 (1.04, 1.27) | 1.05 (0.95, 1.16) | 0.95 (0.86, 1.05) | 0.92 (0.83, 1.02) |
| <4 MLTCs | Ref | Ref | Ref | Ref | Ref | Ref |
| ≥4 MLTCs | 0.73 (0.70, 0.76) | 0.67 (0.65, 0.70) | 0.88 (0.86, 0.91) | 0.75 (0.73, 0.78) | 1.33 (1.29, 1.36) | 1.94 (1.89, 2.00) |

| | First symptom to diagnosis | First loop to diagnosis |
|---|---|---|
| | **Median difference in months (95% CI)** | |
| Male | Ref | Ref |
| Female | 3.3 (2.5, 4.1) | 12.6 (11.6, 13.6) |
| Most affluent | Ref | Ref |
| Most deprived | 8.3 (7.1, 9.6) | 7.5 (5.8, 9.2) |
| White | Ref | Ref |
| South Asian | -3.9 (-6.1, -1.8) | 2.0 (-1.0, 5.0) |
| Black | -7.1 (-10.0, -4.2) | -3.2 (-7.3, 0.9) |
| <4 MLTCs | Ref | Ref |
| ≥4 MLTCs | 18.8 (18.0, 19.6) | 22.3 (21.4, 23.3) |

MLTCs, multiple long-term conditions. NP, natriuretic peptide. Any pathway: at least one of natriuretic peptide (NP) test, echocardiogram or specialist review, recorded in the primary care record in the 6 months prior to HF diagnosis. No pathway: None of NP, echocardiogram or specialist review, recorded in the primary care record in the 6 months prior to HF diagnosis.

**A13 Table: Adjusted group differences in tests, specialist referral, place of diagnosis and lag times, in patients with a recorded indication of heart failure (restricted to 2015-2019; n=80,824)**

| | NP | Echocardiogram | Specialist referral | Any pathway | No pathway | Diagnosis during unplanned hospitalisation |
|---|---|---|---|---|---|---|
| | **OR (95% CI)** | | | | | |
| Male | Ref | Ref | Ref | Ref | Ref | Ref |
| Female | 0.95 (0.90, 0.99) | 0.90 (0.86, 0.93) | 0.85 (0.82, 0.88) | 0.86 (0.83, 0.89) | 1.16 (1.13, 1.20) | 1.20 (1.17, 1.24) |
| Most affluent | Ref | Ref | Ref | Ref | Ref | Ref |
| Most deprived | 0.87 (0.81, 0.93) | 0.77 (0.73, 0.82) | 0.86 (0.82, 0.90) | 0.82 (0.78, 0.86) | 1.23 (1.17, 1.28) | 1.29 (1.23, 1.36) |
| White | Ref | Ref | Ref | Ref | Ref | Ref |
| South Asian | 0.78 (0.68, 0.89) | 0.74 (0.67, 0.83) | 1.15 (1.06, 1.25) | 0.99 (0.92, 1.07) | 1.01 (0.93, 1.09) | 1.16 (1.07, 1.26) |
| Black | 0.68 (0.57, 0.82) | 0.85 (0.74, 0.98) | 1.14 (1.02, 1.27) | 1.01 (0.91, 1.12) | 0.99 (0.89, 1.10) | 1.05 (0.94, 1.17) |
| <4 MLTCs | Ref | Ref | Ref | Ref | Ref | Ref |
| ≥4 MLTCs | 0.68 (0.65, 0.72) | 0.71 (0.68, 0.73) | 0.93 (0.90, 0.96) | 0.78 (0.76, 0.81) | 1.27 (1.23, 1.32) | 1.89 (1.83, 1.95) |

| | First symptom to diagnosis | First loop to diagnosis |
|---|---|---|
| | **Median difference in months (95% CI)** | |
| Male | Ref | Ref |
| Female | 2.6 (2.0, 3.2) | 7.7 (6.8, 8.6) |
| Most affluent | Ref | Ref |
| Most deprived | 1.4 (0.5, 2.2) | 3.7 (2.3, 5.1) |
| White | Ref | Ref |
| South Asian | 1.3 (-0.2, 2.7) | -2.1 (-4.5, 0.4) |
| Black | 0.2 (-1.8, 2.2) | -1.4 (-4.7, 1.9) |
| <4 MLTCs | Ref | Ref |
| ≥4 MLTCs | 16.2 (15.4, 16.9) | 14.6 (13.5, 15.6) |

MLTCs, multiple long-term conditions. NP, natriuretic peptide. Any pathway: at least one NP test, echocardiogram or specialist review, recorded in the primary care record in the 6 months prior to HF diagnosis. No pathway: None of NP, echocardiogram or specialist review, recorded. Adjusted models (n=75,927) included the remainder of sociodemographic characteristics (age sex ethnicity socioeconomic status), year of diagnosis, systolic BP, BMI, cholesterol, smoking and comorbidities (IHD, hypertension, AF, diabetes, CKD, iron deficiency anaemia, stroke, asthma, COPD, cancer, depression, dementia). The exception was the model for MLTCs where the individual comorbidities were removed.

**A14 Figure: Group differences in diagnostic tests, specialist review and diagnosis location**

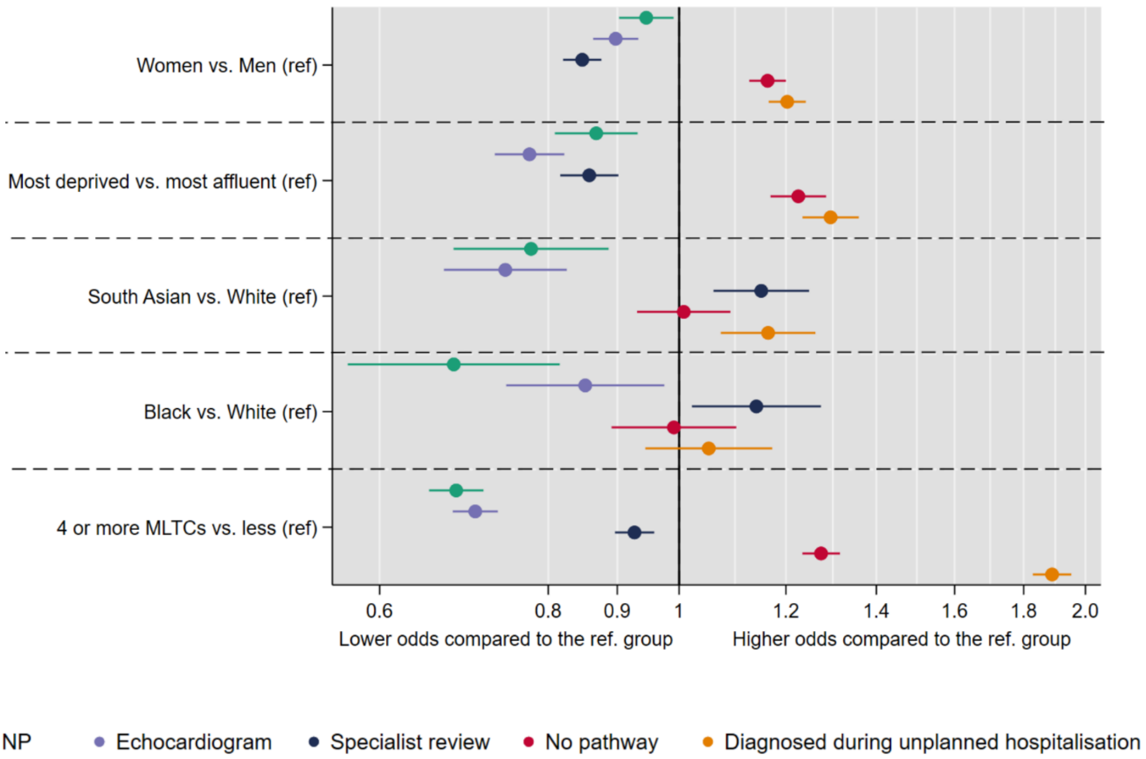


Differences in diagnostic processes, pathways and location of diagnosis by sociodemographic and clinical groups. Models were restricted to patients with a prior indication of heart failure (symptom or loop diuretic use) and diagnosed with HF during the recent years prior to Covid-19 (2015-2019; n=80,824). No pathway: None of NP, echocardiogram or specialist review, recorded in the primary care record in the 6 months prior to HF diagnosis. All models were adjusted for age, sex, ethnicity, socioeconomic status, year of diagnosis, systolic blood pressure, body mass index, cholesterol, smoking, and comorbidities (ischemic heart disease, hypertension, atrial fibrillation, diabetes, chronic kidney disease, iron deficiency anemia, stroke, asthma, chronic obstructive pulmonary disease, cancer, depression, dementia) to estimated adjusted odds ratios with 95% CI. The exception was the model for 4 or more comorbidities where the individual comorbidities were removed.

**A15 Figure: Age adjusted survival in men and women following heart failure diagnosis, by diagnosis group**

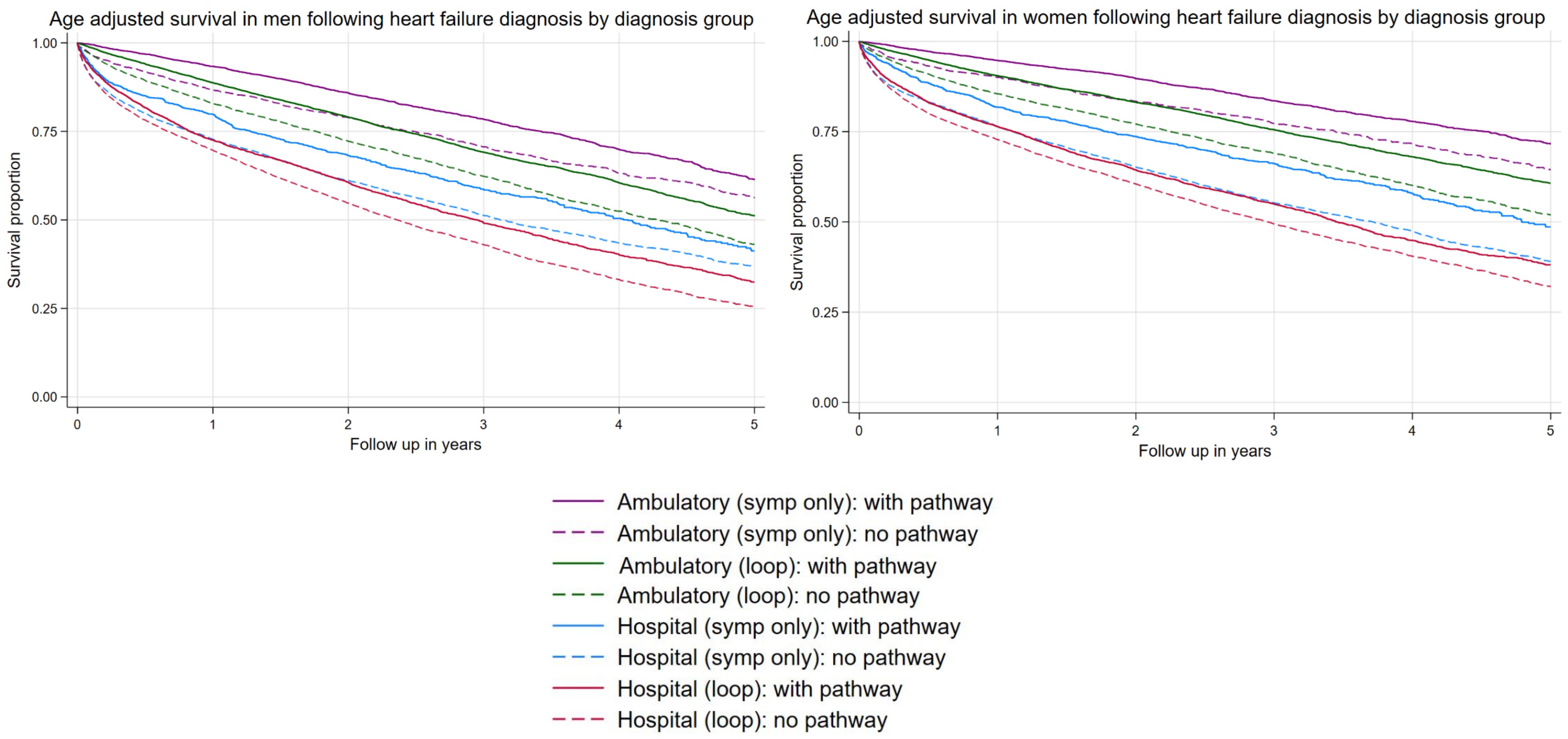


Kaplan-Meier survivor functions for men and women and each diagnostic group, predicted at the mean population age (78 years). Solid lines, groups with primary care-initiated processes prior to diagnosis; Dashed lines, groups without primary care-initiated processes prior to diagnosis.

**A16 Table: Associations between diagnostic pathways and mortality for men and women with heart failure according to location of diagnosis and whether symptoms only or loop diuretic initiation preceded the diagnosis. Data are for 2015-2019 (men=63,127, women=56,228).**

| | **Men** | | **Women** | |
|---|---|---|---|---|
| | Unadjusted | Adjusted* | Unadjusted | Adjusted* |
| | HR (95% CI) | HR (95% CI) | HR (95% CI) | HR (95% CI) |
| **1 year mortality (16,083; 20%)** | | | | |
| Outpatient (prior symptom only): with pathway | Ref | Ref | | |
| Outpatient (prior symptom only): no pathway | 2.2 (1.9, 2.6) | 1.9 (1.7, 2.3) | 2.1 (1.7, 2.5) | 1.9 (1.5, 2.2) |
| Outpatient (prior loop diuretic use): with pathway | 2.2 (2.0, 2.5) | 2.0 (1.7, 2.2) | 2.3 (2.0, 2.7) | 2.1 (1.8, 2.4) |
| Outpatient (prior loop diuretic use): no pathway | 3.6 (3.2, 4.1) | 2.9 (2.5, 3.3) | 4.3 (3.7, 4.9) | 3.4 (2.9, 3.9) |
| Inpatient (prior symptom only): with pathway | 3.6 (3.1, 4.3) | 3.4 (2.9, 4.0) | 4.1 (3.4, 4.8) | 3.6 (3.0, 4.3) |
| Inpatient (prior symptom only): no pathway | 5.4 (4.7, 6.0) | 4.4 (3.9, 5.0) | 5.8 (5.1, 6.7) | 4.5 (3.9, 5.1) |
| Inpatient (prior loop diuretic use): with pathway | 5.9 (5.2, 6.6) | 5.0 (4.4, 5.6) | 5.9 (5.1, 6.8) | 4.8 (4.2, 5.6) |
| Inpatient (prior loop diuretic use): no pathway | 7.2 (6.4, 8.1) | 5.3 (4.7, 6.0) | 7.6 (6.6, 8.6) | 5.3 (4.6, 6.1) |
| **5 year mortality (43,882; 43%)** | | | | |
| Outpatient (prior symptom only): with pathway | Ref | Ref | | |
| Outpatient (prior symptom only): no pathway | 1.4 (1.3, 1.6) | 1.3 (1.2, 1.4) | 1.5 (1.3, 1.7) | 1.4 (1.2, 1.5) |
| Outpatient (prior loop diuretic use): with pathway | 1.8 (1.7, 1.9) | 1.6 (1.5, 1.7) | 2.0 (1.8, 2.1) | 1.7 (1.5, 1.8) |
| Outpatient (prior loop diuretic use): no pathway | 2.5 (2.3, 2.7) | 2.0 (1.8, 2.1) | 3.0 (2.8, 3.2) | 2.3 (2.1, 2.5) |
| Inpatient (prior symptom only): with pathway | 2.2 (2.0, 2.5) | 2.1 (1.9, 2.3) | 2.6 (2.3, 2.9) | 2.2 (2.0, 2.5) |
| Inpatient (prior symptom only): no pathway | 2.9 (2.7, 3.1) | 2.5 (2.3, 2.6) | 3.7 (3.4, 4.0) | 2.8 (2.6, 3.1) |
| Inpatient (prior loop diuretic use): with pathway | 3.4 (3.2, 3.7) | 2.8 (2.6, 3.1) | 3.9 (3.6, 4.3) | 3.1 (2.8, 3.4) |
| Inpatient (prior loop diuretic use): no pathway | 4.4 (4.1, 4.7) | 3.2 (3.0, 3.4) | 4.9 (4.6, 5.3) | 3.3 (3.1, 3.6) |

Any pathway: at least one of natriuretic peptide (NP) test, echocardiogram or specialist review, recorded in the primary care record in the 6 months prior to HF diagnosis. No pathway: None of NP, echocardiogram or specialist review, recorded in the primary care record in the 6 months prior to HF diagnosis. Models were restricted to patients with a prior indication of heart failure (symptom or loop diuretic use) and diagnosed with HF during the recent years prior to Covid-19 (2015-2019; n=80,824). Adjusted models (n=75,927) included age sex ethnicity socioeconomic status, year of diagnosis, systolic blood pressure, body mass index, cholesterol, smoking and comorbidities (ischaemic heart disease, hypertension, atrial fibrillation, diabetes, chronic kidney disease, iron deficiency anaemia, stroke, asthma, chronic obstructive pulmonary disease, cancer, depression, dementia) and prescribed drugs at diagnosis (beta blocker, RAAS, MRA, ARNi, SGLT2i)..

**A17 Table: 1 year mortality comparison among all patients versus those remaining after excluding individuals with only a primary care code and no diagnostic test or GRMT prescription**

| | All patients | Excluding individuals with only a primary care code and no diagnostic test or GRMT prescription (n=17,604), |
|---|---|---|
| | Adjusted HR (95% CI) | |
| Outpatient (prior symptom only): with pathway | Ref | Ref |
| Outpatient (prior symptom only): no pathway | 1.9 (1.7, 2.2) | 1.9 (1.7, 2.2) |
| Outpatient (prior loop diuretic use): with pathway | 2.0 (1.8, 2.2) | 2.0 (1.8, 2.2) |
| Outpatient (prior loop diuretic use): no pathway | 3.1 (2.8, 3.4) | 2.8 (2.5, 3.1) |
| Inpatient (prior symptom only): with pathway | 3.5 (3.1, 3.9) | 3.5 (3.1, 3.9) |
| Inpatient (prior symptom only): no pathway | 4.4 (4.0, 4.9) | 4.5 (4.1, 4.9) |
| Inpatient (prior loop diuretic use): with pathway | 4.9 (4.4, 5.4) | 4.9 (4.4, 5.4) |
| Inpatient (prior loop diuretic use): no pathway | 5.3 (4.8, 5.8) | 5.4 (4.9, 5.9) |

Data are presented as median (IQR) for continuous measures, and n (%) for categorical measures.

**A18 Table: Adjusted group differences in tests, specialist referral and place of diagnosis, taking account of variation between general practices (restricted to 2015-2019; n=80,824)**

| | NP | Echocardiogram | Specialist referral | Any pathway | No pathway | Diagnosis during unplanned hospitalisation |
|---|---|---|---|---|---|---|
| | **Adjusted Group differences (OR 95% CI) including further adjustment for practice variation** | | | | | |
| Male | Ref | Ref | Ref | Ref | Ref | Ref |
| Female | 0.91 (0.87, 0.96) | 0.85 (0.81, 0.89) | 0.83 (0.81, 0.86) | 0.84 (0.82, 0.87) | 1.19 (1.15, 1.22) | 1.21 (1.17, 1.25) |
| Most affluent | Ref | Ref | Ref | Ref | Ref | Ref |
| Most deprived | 0.98 (0.89, 1.08) | 0.89 (0.81, 0.97) | 0.81 (0.76, 0.86) | 0.80 (0.75, 0.85) | 1.25 (1.18, 1.33) | 1.24 (1.17, 1.31) |
| White | Ref | Ref | Ref | Ref | Ref | Ref |
| South Asian | 0.96 (0.82, 1.14) | 0.86 (0.75, 1.00) | 1.03 (0.94, 1.13) | 0.97 (0.88, 1.06) | 1.03 (0.94, 1.13) | 1.14 (1.04, 1.24) |
| Black | 0.82 (0.66, 1.02) | 0.94 (0.78, 1.13) | 0.99 (0.87, 1.11) | 0.94 (0.83, 1.06) | 1.06 (0.95, 1.20) | 1.05 (0.93, 1.18) |
| <4 MLTCs | Ref | Ref | Ref | Ref | Ref | Ref |
| ≥4 MLTCs | 0.63 (0.59, 0.66) | 0.64 (0.61, 0.67) | 0.92 (0.89, 0.95) | 0.78 (0.75, 0.80) | 1.29 (1.25, 1.33) | 1.90 (1.83, 1.96) |
| ICC for GP practice variation | 0.51 (0.48, 0.54) | 0.74 (0.71, 0.76) | 0.08 (0.07, 0.09) | 0.08 (0.07, 0.09) | 0.08 (0.08, 0.09) | 0.04 (0.03, 0.04) |

MLTCs, multiple long-term conditions. NP, natriuretic peptide. Any pathway: at least one of natriuretic peptide (NP) test, echocardiogram or specialist review, recorded in the primary care record in the 6 months prior to HF diagnosis. No pathway: None of NP, echocardiogram or specialist review, recorded in the primary care record in the 6 months prior to HF diagnosis. Adjusted models (n=75,927) included the remainder of sociodemographic characteristics (age sex ethnicity socioeconomic status), year of diagnosis, systolic blood pressure, body mass index, cholesterol, smoking and comorbidities (ischaemic heart disease, hypertension, atrial fibrillation, diabetes, chronic kidney disease, iron deficiency anaemia, stroke, asthma, chronic obstructive pulmonary disease, cancer, depression, dementia). The exception was the model for 4 or more comorbidities where the individual comorbidities were removed. GP practice included in the model as a random effect.

**A19 Table: Baseline characteristics comparing all patients with those that did not survive their index date**

| | All patients<br>**N=408,306** | Died on their index date<br>**N=3,867** | |
|---|---|---|---|
| Age at diagnosis (ears) | 78.0 (69.0-85.0) | 80.0 (71.0-87.0) | |
| Female | | 192,340 (47%) | 1,835 (47%) |
| Diagnosis as inpatient | 180,384 (44%) | 903 (23%) | |
| IMD level 1 (affluent) | 78,752 (19%) | 638 (17%) | |
| IMD level 5 (deprived) | 81,752 (20%) | 859 (22%) | |
| Smoker | 83,319 (21%) | 1,004 (28%) | |
| Ischaemic heart disease | 196,787 (48%) | 1,885 (49%) | |
| Myocardial infarction | 159,092 (39%) | 1,180 (31%) | |
| Hypertension | 286,429 (70%) | 2,346 (61%) | |
| Atrial fibrillation | 160,483 (39%) | 931 (24%) | |
| Obesity | 119,042 (29%) | 716 (19%) | |
| Diabetes | 110,422 (27%) | 879 (23%) | |
| CKD | 160,787 (39%) | 1,517 (39%) | |
| Iron deficiency anaemia | 53,276 (13%) | 438 (11%) | |
| Stroke | 49,823 (12%) | 553 (14%) | |
| Asthma | 87,589 (21%) | 735 (19%) | |
| COPD | 81,245 (20%) | 768 (20%) | |
| Cancer | 110,365 (27%) | 956 (25%) | |
| Depression | 51,231 (13%) | 497 (13%) | |
| Dementia | 21,702 (5%) | 333 (9%) | |
| Beta blocker | 90,632 (22%) | 328 (8%) | |
| ACEi | 147,170 (36%) | 1,040 (27%) | |
| ARB | 55,838 (14%) | 279 (7%) | |
| Loop diuretic | 154,965 (38%) | 1,177 (30%) | |

Data are presented as median (1st, 3rd quartiles) for continuous measures, and n (%) for categorical measures. CKD, chronic kidney disease; eGFR, estimated glomerular filtration rate; COPD, chronic obstructive pulmonary disease, ACEi, angiotensin converting enzyme inhibitor; ARB, angiotensin II receptor blockers; MRA; mineralocorticoid receptor antagonist, ARNi; angiotensin receptor/neprilysin inhibitor, SGLT2i; sodium/glucose cotransporter 2 inhibitor. Prescribed drugs were identified by at least one prescription in a 6-month time window prior to heart failure diagnosis.

**A20 Table: Associations between diagnostic pathways and mortality for patients with heart failure according to location of diagnosis and whether symptoms only or loop diuretic initiation preceded the diagnosis. Data are for 2015-2019 (n=80,824). Mortality models comparing all patients with those that survived their index date (n=80,611).**

| | All patients Adjusted HR (95% CI) | Survived their index date Adjusted HR (95% CI) |
|---|---|---|
| Outpatient (prior symptom only): with pathway | Ref | Ref |
| Outpatient (prior symptom only): no pathway | 1.9 (1.7, 2.2) | 1.8 (1.6, 2.0) |
| Outpatient (prior loop diuretic use): with pathway | 2.0 (1.8, 2.2) | 2.0 (1.8, 2.2) |
| Outpatient (prior loop diuretic use): no pathway | 3.1 (2.8, 3.4) | 3.0 (2.7, 3.3) |
| Inpatient (prior symptom only): with pathway | 3.5 (3.1, 3.9) | 3.4 (3.0, 3.9) |
| Inpatient (prior symptom only): no pathway | 4.4 (4.0, 4.9) | 4.4 (4.0, 4.8) |
| Inpatient (prior loop diuretic use): with pathway | 4.9 (4.4, 5.4) | 4.8 (4.4, 5.3) |
| Inpatient (prior loop diuretic use): no pathway | 5.3 (4.8, 5.8) | 5.2 (4.8, 5.7) |

Any pathway: at least one of natriuretic peptide (NP) test, echocardiogram or specialist review, recorded in the primary care record in the 6 months prior to HF diagnosis. No pathway: None of NP, echocardiogram or specialist review, recorded in the primary care record in the 6 months prior to HF diagnosis. Models were restricted to patients with a prior indication of heart failure (symptom or loop diuretic use) and diagnosed with HF during the recent years prior to Covid-19 (2015-2019). Adjusted models included age sex ethnicity socioeconomic status, year of diagnosis, systolic blood pressure, body mass index, cholesterol, smoking and comorbidities (ischaemic heart disease, hypertension, atrial fibrillation, diabetes, chronic kidney disease, iron deficiency anaemia, stroke, asthma, chronic obstructive pulmonary disease, cancer, depression, dementia) and prescribed drugs at diagnosis (beta blocker, RAAS, MRA, ARNi, SGLT2i).
